# Identifying the optimal rapid antigen test for screening and determining the end of isolation: A modeling study

**DOI:** 10.1371/journal.pcbi.1013102

**Published:** 2026-04-02

**Authors:** Yong Dam Jeong, William S. Hart, Masahiro Ishikane, Kwang Su Kim, Jong Hyuk Byun, Il Hyo Jung, Montie T. Harrison, Kazuyuki Aihara, Norio Ohmagari, Christopher B. Brooke, Ruian Ke, Robin N. Thompson, Shingo Iwami

**Affiliations:** 1 Interdisciplinary Biology Laboratory (iBLab), Division of Natural Science, Graduate School of Science, Nagoya University, Nagoya, Japan; 2 Department of Applied Mathematics, Pukyong National University, Busan, South Korea; 3 Mathematical Institute, University of Oxford, Oxford, United Kingdom; 4 Disease Control and Prevention Centre, National Centre for Global Health and Medicine, Japan Institute for Health Security, Tokyo, Japan; 5 Department of Science System Simulation, Pukyong National University, Busan, South Korea; 6 Department of Mathematics and Institute of Mathematical Sciences, Pusan National University, Busan, South Korea; 7 Institute for Future Earth, Pusan National University, Busan, South Korea; 8 International Research Center for Neurointelligence, The University of Tokyo Institutes for Advanced Study, The University of Tokyo, Tokyo, Japan; 9 Department of Microbiology, University of Illinois at Urbana-Champaign, Urbana, Illinois, United States of America; 10 Theoretical Biology and Biophysics, Los Alamos National Laboratory, Los Alamos, New Mexico, United States of America; 11 Institute of Mathematics for Industry, Kyushu University, Fukuoka, Japan; 12 Institute for the Advanced Study of Human Biology (ASHBi), Kyoto University, Kyoto, Japan; 13 Interdisciplinary Theoretical and Mathematical Sciences Program (iTHEMS), RIKEN, Saitama, Japan; 14 NEXT-Ganken Program, Japanese Foundation for Cancer Research (JFCR), Tokyo, Japan; 15 Science Groove Inc., Fukuoka, Japan; Pennsylvania State University, UNITED STATES OF AMERICA

## Abstract

During the COVID-19 pandemic, rapid antigen tests (RATs) were used to detect infections, improving the effectiveness of targeted non-pharmaceutical interventions (NPIs). However, RATs based on either nasal swab or saliva samples were used, raising the question as to which type of RAT is most effective at detecting viral infections. Here, we develop a model-driven computational framework to assess different RATs and identify the most suitable test for a specified purpose, such as infection screening or determining the end of isolation, for various viral infections. Using symptomatic COVID-19 cases as a case study, we found that saliva-based RATs reduced transmission risk on average by 6.2% (95% CI: 6.1 – 6.3) compared to nasal-based RATs in the pre-symptomatic period. In addition, by ending isolation of infected individuals who have developed symptoms when consecutive RATs return negative results, the mean risk of transmission was reduced by 5.9% (95% CI: 5.7 – 6.1) using a saliva-based RAT compared to using a nasal-based RAT. These findings suggest that saliva RATs may be a useful option for mitigating SARS-CoV-2 transmission effectively. However, real-world variability in test sensitivity and sample collection should be carefully considered when evaluating the practical use of each RAT type. Our novel approach can be applied to other viruses and types of tests, enabling its use to inform public health policy decisions about which types of RAT to prioritize in future infectious disease epidemics.

## Author summary

Rapid antigen tests (RATs) have been widely used to prevent SARS-CoV-2 transmission, yet debates persist regarding which sample type yields the most effective results. In this study, we develop a model-driven computational framework to evaluate the comparative effectiveness of RATs that use different sample types. Our findings suggest that saliva RATs are more likely to mitigate SARS-CoV-2 transmission than nasal RATs, providing empirical evidence for the broader use of saliva-based tests in managing COVID-19 outbreaks. However, when assessing the practical application of each RAT type, real-world variability in test sensitivity and sample collection procedures should be carefully considered. The flexible design of our framework also enables its adaptation to the evaluation of other diagnostic tests and various viral infectious diseases. As such, our approach could offer a valuable tool for informing public health policy decisions, both for the ongoing management of COVID-19 and for responding to future outbreaks of emerging infectious diseases.

## Introduction

Testing plays a critical role in preventing the spread of viral infections. If infected individuals are detected quickly and diagnosed accurately, measures such as isolation can be taken to mitigate transmission. Reverse transcription polymerase chain reaction (RT-PCR) testing has long been considered the gold standard for detecting viral RNA, and has high sensitivity (i.e., even low levels of the virus can be detected). However, rapid antigen tests (RATs) provide an alternative approach for detecting viral infections. For example, RATs were developed to detect influenza viral nucleoprotein antigens, and have been widely used to diagnose influenza cases [1], in part due to the speed at which test results are obtained. Since the emergence of SARS-CoV-2, a multiplex PCR to amplify different RNA sequences simultaneously has been utilized to identify influenza and SARS-CoV-2 infections [2]. However, RATs continue to be recommended [3] to identify SARS-CoV-2 infections due to a range of advantages (e.g., shorter turnaround time, lower cost, and easier accessibility), even though their sensitivity is lower than that of RT-PCR tests.

RATs use a variety of swab specimen types for different infectious diseases: nasal swab or saliva specimens for respiratory infectious diseases [4,5] and skin lesion or vaginal swabs for sexually transmitted diseases (STD). In response to COVID-19, RATs have often involved nasal swab specimens, as previous studies have indicated that nasal swab-based RATs have high sensitivity [6]. However, an alternative option is saliva-based RATs, which are less invasive and therefore have the potential to lead to improved uptake in the host population [7]. While saliva-based RATs have been the focus of many previous studies [8–14], the difference in effectiveness between nasal swab and saliva specimens remains a topic of debate.

Targeted non-pharmaceutical interventions (NPIs) can be more effective if case detection is improved using RT-PCR tests or RATs [5]. Early in the COVID-19 pandemic, symptom-based screening was widely used to reduce transmission [15,16]. However, since transmission of SARS-CoV-2 from individuals who are not displaying symptoms is commonplace [17–20], it was vital to detect asymptomatic and pre-symptomatic cases. Testing-based surveillance has thus played a crucial role in limiting SARS-CoV-2 transmission [21–23]. Along with testing, isolation has been an effective way of responding to COVID-19 [24,25], and more recently mpox [26]. Widely used guidelines have involved isolating infected individuals for a fixed time period for COVID-19 (e.g., until 10 days have elapsed since symptoms first appeared) [27] and using a symptom-based criterion for mpox (e.g., until skin lesions have resolved) [28]. However, such strategies do not fully account for individual variability in viral loads, and thus might not prevent secondary transmission entirely. As an alternative, testing-based guidelines have been adopted, which involve ending isolation following (typically) successive negative RT-PCR or RAT results [27,29,30]. Different testing protocols can be applied for different infectious diseases, but irrespective of the scenario under consideration it is important to analyze and evaluate the effectiveness of different tests at preventing transmission.

A substantial modeling literature on COVID-19 has shown that, for screening, test frequency and turnaround time outweigh marginal gains in assay sensitivity [21,22], and that combining testing with optimized quarantine can both reduce transmission and safely shorten isolation [31–33], and has compared policy packages across campus, contact tracing, and travel settings. Furthermore, in our recent studies, we optimized screening strategies using RATs [34] and developed guidance for ending isolation contingent on consecutive negative RAT results [25]. These studies typically assumed a single specimen type without considering saliva-based testing and often mapped viral load to infectiousness via binary thresholds. However, head-to-head comparisons of nasal- versus saliva-based RATs under distinct public-health objectives – pre-symptomatic screening and post-symptomatic de-isolation – remain limited, and few analyses explicitly link paired within-host dynamics across different sites to a continuous infectiousness profile calibrated by cell culture data on viral replication.

In this study, we propose a unified framework to analyze and compare test performance across objectives. Using SARS-CoV-2 as a case study, we fit a within-host model to individual-level longitudinal viral load data from paired nasal and saliva samples to estimate site-specific dynamics. We then translate viral RNA load into time-varying infectiousness and assess control strategies using nasal swab and saliva-based RATs for two different goals: (i) screening to identify infections in the pre-symptomatic phase (i.e., identifying cases before symptom onset) and (ii) testing to allow post-symptomatic individuals to stop isolating (i.e., releasing infected individuals based on successive negative test results after symptom onset). This novel computational framework quantifies the effectiveness of different tests in preventing transmission and can be used to inform public health policy for newly emerging infectious diseases.

## Materials and methods

### Study data

We collected longitudinal viral load data for paired nasal swab and saliva samples from symptomatic COVID-19 cases by undertaking a literature search using PubMed. Specifically, we used the following query: (“SARS-CoV-2” or “COVID-19”) and (“viral load” or “Ct value”) and (“paired” or “saliva” or “nasal” or “nasopharyngeal”) and (“longitudinal” or “symptom onset”). We then reviewed each article identified in the search to extract the relevant data based on the following inclusion criteria: 1) timing of symptom onset was recorded; 2) viral load was measured at least at three different time points; 3) cases had paired nasal (or nasopharyngeal) and saliva samples; and 4) cases did not receive any antivirals (since antiviral treatment is not considered in our model). A total of 2 publications satisfied those criteria, and 58 cases were identified [35,36]. Those cases were confirmed between 2020 and 2021. We used only de-identified data from published studies and thus ethics approval was not required. Details are summarized in S1 Table.

### Within-host viral dynamics model of SARS-CoV-2 and parameter estimation

We used a previously proposed model of SARS-CoV-2 viral dynamics, given by [23,25]:


$$\begin{array}{c} \frac{df_{k}}{dt}=-b_{k}f_{k}V_{k},\\ \;\\ \frac{dV_{k}}{dt}=\gamma_{k}f_{k}V_{k}-\delta_{k}V_{k}, \end{array}$$ (1)

where $f_{k}(t)$ and $V_{k}(t)$ (k=n,s) represent the fraction of uninfected target cells and viral RNA load (copies/ml) in a site k at time t, respectively. Note that t=0 means the time of infection, so $f_{k}(0)=1$. The initial viral load was assumed as $V_{k}(0)=0.01$ (copies/ml) [25]. The parameters $b_{k}$, $\gamma_{k}$, and $\delta_{k}$ (k=n,s) are the rate constant for virus infection, the maximum viral replication rate, and the death rate of infected cells, respectively. The indices n and s indicate viral dynamics in nasal swab and saliva samples, respectively.

We estimated the model parameters by fitting the model to the paired viral load data. Because the timing of symptom onset was recorded for each patient, the incubation period (i.e., the number of days between infection and symptom onset), $t_{s}$, was also estimated. A nonlinear mixed-effects (NLME) model was employed for parameter estimation [24,25]. This approach captures the heterogeneity in the SARS-CoV-2 viral dynamics by including both a fixed effect (common features in the population, i.e., population parameter) and a random effect (different features between individuals) in each parameter. Population and individual parameters were estimated using Stochastic Approximation Expectation Maximization (SAEM) [37] and Markov Chain Monte Carlo (MCMC) methods. The SAEM algorithm computes an approximation of the maximum likelihood estimator of the parameters, assuming a normal distribution (mean 0 and variance $\sigma^{\text{2}}$) for the residuals (i.e., differences between predicted log viral load and measured log viral load) to quantify the error in viral load measurements. Distributional estimates of individual parameter values were subsequently computed using MCMC, and best-fit Empirical Bayes Estimates (EBEs) were then calculated for each individual. Estimation was undertaken using MONOLIX 2024R1 (www.lixoft.com). The estimated viral dynamics parameter values are summarized in S2 Table.

To account for individual variability in viral dynamics when assessing the effect of RATs on reducing SARS-CoV-2 transmission, we simulated viral loads in nasal swab and saliva samples, $V_{n}(t)$ and $V_{s}(t)$, for N=10,000 virtual infected individuals using the viral dynamics model. Viral dynamics parameters and the incubation period were sampled for each virtual patient from estimated model parameter distributions. To quantify uncertainty (i.e., confidence intervals) in the target outcomes (described in the following subsections) while keeping the sample size fixed, we applied a full-size bootstrap of the 10,000 virtual individuals. In each of 100 iterations, we resampled 10,000 individuals with replacement, recomputed all outcomes, and summarized their distribution across iterations.

### Modelling transmissibility of SARS-CoV-2

To quantify transmissibility, we first calculated the transmission probability (i.e., the probability of virus transmission occurring in a typical contact), as described in [38]. For this, the relationship between viral load and infectious viruses is first considered. It is assumed that the number of infectious viruses from a cell culture experiment is a random variable, Y, that follows a Poisson distribution. The mean number of infectious viruses, E(Y), is denoted by $V_{inf}$. Here, it is assumed that $V_{\inf}$ is related to the viral load, V, via a Hill function as follows:


$$E(Y)=V_{inf}=V_{m}\frac{V^{h}}{V^{h}+\overset{h}{\underset{m}{K}}},$$

where $V_{m}$, $K_{m}$, and h are the maximum mean number of infectious viruses, the half-saturation constant, and the Hill coefficient, respectively. If each infectious virus independently establishes infection in the cell culture with a probability ρ, then the number of viruses resulting in infection follows a Poisson distribution with parameter $\lambda_{\text{1}}={\rho V}_{inf}$. Thus, the following probability of cell culture positivity, $p_{+}$, can be obtained:


$$\begin{array}{c} p_{+}=1-\exp\left(-\lambda_{\text{1}}\right)=1-\exp\left(-{\rho V}_{inf}\right)=1-\exp\left(-\phi \frac{V^{h}}{V^{h}+\overset{h}{\underset{m}{K}}}\right), \end{array}$$ (2)

where $\phi =\rho V_{m}$. The parameters were estimated using a nonlinear least squares method to fit Eq. (2) to reported values of the probability of cell culture positivity [39], as depicted in S1 Fig.

During each contact between an infected individual and a susceptible individual, the average total number of infectious viruses reaching the susceptible individual, $\lambda_{\text{2}}=\xi V_{inf}$, is assumed to be proportional to the mean number of infectious viruses, $V_{inf}$, collected in a cell culture experiment (which is itself a fraction of the total infectious viral load), where ξ is a constant of proportionality. For respiratory viruses, viral particles from the infected individual tend to be randomly distributed in the air [40], so the number of infectious viruses reaching the susceptible individual during the contact is assumed to be a random variable, X, which follows a Poisson distribution with parameter $\lambda_{\text{2}}$. Further, it is assumed that the number of infectious viruses to successfully establish infection after reaching the susceptible individual is a random variable, Z, that follows a binomial distribution with X trials and success probability ν, conditional on the value of X. Thus, Z follows a Poisson distribution with parameter $\lambda_{\text{3}}=\nu \lambda_{\text{2}}=\nu \xi V_{inf}=\theta \frac{V^{h}}{V^{h}+\overset{h}{\underset{m}{K}}},$ where $\theta =\nu \xi V_{m}$. Finally, the per-contact transmission probability for an infected individual over a short contact at time t is given by


$$\begin{array}{c} p(t)=1-\exp\left(-\theta \frac{{V(t)}^{h}}{{V(t)}^{h}+\overset{h}{\underset{m}{K}}}\right), \end{array}$$ (3)

where 1−exp(−θ) is the maximum transmission probability at very high viral loads (this maximum transmission probability is approximately θ for small value of θ). Additionally, we can consider an overall transmission probability incorporating possible transmission routes from various sites. Assuming a set of events of transmission from M different sites, $\overset{M}{\underset{k=1}{\left\{E_{k}\right\}}}$ is mutually independent, we can compute the overall transmission probability as follows:

$\begin{array}{c} P\left(\overset{M}{\underset{k=1}{⋃}}E_{k}\right)=1-\prod_{k=1}^{M}\left(1-P\left(E_{k}\right)\right)=1-\prod_{k=1}^{M}\left(1-p_{k}\right)=1-\prod_{k=1}^{M}\left[\exp\left(-\theta_{k}\frac{\overset{h}{\underset{k}{V}}}{\overset{h}{\underset{k}{V}}+\overset{h}{\underset{m}{K}}}\right)\right], \end{array}$

where $V_{k}$ corresponds to the viral load at a sitek. Thus, the overall transmission probability of an infected individual at timet, p(t), is defined as


$$p(t)=1-\prod_{k=1}^{M}\left[\exp\left(-\theta_{k}\frac{\overset{h}{\underset{k}{V}}}{\overset{h}{\underset{k}{V}}+\overset{h}{\underset{m}{K}}}\right)\right]=1-\exp\left(-\sum_{k=1}^{M}\theta_{k}\frac{\overset{h}{\underset{k}{V}}}{\overset{h}{\underset{k}{V}}+\overset{h}{\underset{m}{K}}}\right).$$

In our analysis, we considered the overall transmission of SARS-CoV-2 incorporating viral dynamics in nasal swab and saliva samples (i.e., M=2), since the transmission of respiratory viruses is generally caused by droplets from nasal and oral cavities [41]. Then, the overall transmission probability of a SARS-CoV-2 infected individual during a short-duration contact at time t is


$$\begin{array}{c} p(t)=1-exp\left[-\left(\theta_{n}\frac{{V_{n}(t)}^{h}}{{V_{n}(t)}^{h}+\overset{h}{\underset{m}{K}}}+\theta_{s}\frac{{V_{s}(t)}^{h}}{{V_{s}(t)}^{h}+\overset{h}{\underset{m}{K}}}\right)\right], \end{array}$$ (4)

where $V_{n}$ and $V_{s}$ indicate reconstructed viral loads in nasal and oral cavities, respectively. Note that $1-\exp\left(-\left(\theta_{n}+\theta_{s}\right)\right)$ is the maximum transmission probability, similarly to Eq. (3). In our analysis, it was assumed that $\theta_{n}=\theta_{s}$, denoted by θ, since θ represents per-contact exposure shared by both routes within the same encounter, whereas route-specific differences are captured by the Hill-type saturation terms $\frac{{V_{k}(t)}^{h}}{{V_{k}(t)}^{h}+\overset{h}{\underset{m}{K}}}\;(k\in \{n,s\})$. Under short-duration contacts, the per-contact transmission probability is small (rare-event condition, θ≪1), supported by prospective contact-tracing analyses [42,43]. Using a first-order Taylor approximation, we can obtain


$$\begin{array}{c} p(t)\approx \theta \times \left(\frac{{V_{n}(t)}^{h}}{{V_{n}(t)}^{h}+\overset{h}{\underset{m}{K}}}+\frac{{V_{s}(t)}^{h}}{{V_{s}(t)}^{h}+\overset{h}{\underset{m}{K}}}\right). \end{array}$$

The infectiousness at time t since infection is then given by


$$\begin{array}{c} \beta (t)=c\times p(t), \end{array}$$ (5)

where c is the contact rate, and substituting the approximation gives


$$\begin{array}{c} \beta (t)\approx C\times \left(\frac{{V_{n}(t)}^{h}}{{V_{n}(t)}^{h}+\overset{h}{\underset{m}{K}}}+\frac{{V_{s}(t)}^{h}}{{V_{s}(t)}^{h}+\overset{h}{\underset{m}{K}}}\right), \end{array}$$ (6)

where C (=c×θ) is a constant. As an illustrative example, the infectiousness, β(t), is shown in S2B Fig, based on estimated viral load dynamics in nasal swab and saliva samples under the best-fit population parameters (S2A Fig).

Then, a basic reproduction number (i.e., expected number of secondary cases), $R_{\text{0}}$, is defined by the total infectiousness over time since infection [44,45]:


$$\begin{array}{c} R_{\text{0}}=\int_{\text{0}}^{\infty }\hat{\beta }(\tau )d\tau =\int_{\text{0}}^{t_{s}}\hat{\beta }(\tau )d\tau +\int_{t_{s}}^{\infty }\hat{\beta }(\tau )d\tau =\overset{pre}{\underset{\text{0}}{R}}+\overset{post}{\underset{\text{0}}{R}}, \end{array}$$ (7)

where $\overset{pre}{\underset{\text{0}}{R}}$ and $\overset{post}{\underset{\text{0}}{R}}$ represent the expected number of secondary cases generated by pre-symptomatic and post-symptomatic transmission, respectively. In our analysis, the basic reproduction number was assumed to be a mean of N individual-specific reproduction numbers, $R_{i}$ (i.e., the expected number of secondary cases from an infected individual i), as follows:


$$\begin{array}{c} R_{\text{0}}=\frac{\text{1}}{N}\sum_{i=1}^{N}R_{i}=\frac{\text{1}}{N}\sum_{i=1}^{N}\left(\int_{\text{0}}^{\infty }\beta_{i}(\tau )d\tau \right)=\frac{\text{1}}{N}\sum_{i=1}^{N}\left(\int_{\text{0}}^{t_{s,i}}\beta_{i}(\tau )d\tau +\int_{t_{s,i}}^{\infty }\beta_{i}(\tau )d\tau \right)=\frac{\text{1}}{N}\sum_{i=1}^{N}\left(\overset{pre}{\underset{i}{R}}+\overset{post}{\underset{i}{R}}\right), \end{array}$$ (8)

where $\beta_{i}(\tau )=C\times \left(\frac{{V_{n,i}(\tau )}^{h}}{{V_{n,i}(\tau )}^{h}+\overset{h}{\underset{m}{K}}}+\frac{{V_{s,i}(\tau )}^{h}}{{V_{s,i}(\tau )}^{h}+\overset{h}{\underset{m}{K}}}\right)$ and $t_{s,i}$ indicate the infectiousness profile and the incubation period of individual i, respectively. $V_{n,i}$ and $V_{s,i}$ correspond to the reconstructed nasal and saliva viral loads of individuali, respectively. $\overset{pre}{\underset{i}{R}}$ and $\overset{post}{\underset{i}{R}}$ are the expected number of secondary cases during the pre-symptomatic and post-symptomatic phases from the individual i, respectively. Eq. (8) was used to determine the scaling constant, C, under a specific value of the basic reproduction number (i.e., $R_{\text{0}}=3.0$) [46,47]. To represent different epidemic contexts, we also recalibrated C to alternative $R_{\text{0}}$ values (1.5−6.0). All parameter values are summarized in S3 Table.

Further, we defined a risk of transmission, that is, a probability that an individual generates at least one transmission during the infectious period. Here, it was assumed that an individual i generates Poisson-distributed numbers of pre-symptomatic and post-symptomatic transmissions with means $\overset{pre}{\underset{i}{R}}$and $\overset{post}{\underset{i}{R}}$. Thus, the risk of transmission during the pre-symptomatic and post-symptomatic phases from the individual i can be defined by


$$\begin{array}{c} \begin{array}{c} \overset{pre}{\underset{i}{r}}=1-exp(-\overset{pre}{\underset{i}{R}}),\\ \;\\ \overset{post}{\underset{i}{r}}=1-\exp\left(-\overset{post}{\underset{i}{R}}\right). \end{array} \end{array}$$ (9)

Then, by averaging over the population (of size N), we can obtain an overall risk of transmission during the pre-symptomatic and post-symptomatic phases:


$$\begin{array}{c} \begin{array}{c} r^{pre}=\frac{\text{1}}{N}\sum_{i=1}^{N}\overset{pre}{\underset{i}{r}}=\frac{\text{1}}{N}\sum_{i=1}^{N}1-exp(-\overset{pre}{\underset{i}{R}}),\\ \;\\ r^{post}=\frac{\text{1}}{N}\sum_{i=1}^{N}\overset{post}{\underset{i}{r}}=\frac{\text{1}}{N}\sum_{i=1}^{N}1-exp(-\overset{post}{\underset{i}{R}}). \end{array} \end{array}$$ (10)

Additionally, to assess the impact of overdispersion in the offspring distribution, we replaced the Poisson assumption with a negative binomial distribution with the mean $R_{i}$ and the dispersion parameter k. Under this parameterization, the probability that individual i causes at least one secondary infection during a given phase is $1-{\left(1+R_{i}/k\right)}^{-k}\;,$ which reduces to the Poisson expression $1-exp\left(-R_{i}\right)$ as k→∞. Here, we fixed k=0.41, consistent with the estimate of overdispersion in COVID-19 transmission [48,49].

### Probability of detection by RAT

An infected individual can be detected by a RAT if a positive test result is obtained, that is, a measured viral load exceeds a limit of detection. We thus considered that for a test taken by individual i at time since infection t, detection occurs with the following probabilities [24,25]:


$$\overset{n}{\underset{d,i}{p}}(t)=P\left({\hat{V}}_{n,i}(t)\geq {LOD}_{n}\right)=P\left(\log_{10}{\hat{V}}_{n,i}(t)\geq \log_{10}{LOD}_{n}\right)\;for\;nasal\;RAT,$$

$$\overset{s}{\underset{d,i}{p}}(t)=P\left({\hat{V}}_{s,i}(t)\geq {LOD}_{s}\right)=P\left(\log_{10}{\hat{V}}_{s,i}(t)\geq \log_{10}{LOD}_{s}\right)\;for\;saliva\;RAT,$$

where ${\hat{V}}_{n,i}$ and ${\hat{V}}_{s,i}$ are measured viral loads for the individual i by nasal and saliva RATs, respectively, which are assumed to be normally distributed on log scales such that $\log_{10}{\hat{V}}_{n,i}\sim N(\log_{10}V_{n,i},\overset{\text{2}}{\underset{n}{\sigma }})$ and $\log_{10}{\hat{V}}_{s,i}\sim N(\log_{10}V_{s,i},\overset{\text{2}}{\underset{s}{\sigma }})$. The measurement errors for nasal and saliva RATs ($\sigma_{n}$ and $\sigma_{s}$) were estimated in the parameter estimation process, and the values are described in S2 Table. Here, ${LOD}_{n}$ and ${LOD}_{s}$ represent the limit of detection for nasal and saliva RATs. These values were both assumed to be 6.0 log10 copies/ml as a baseline [50–52], and sensitivity analyses were performed (from 3 to 7 log10 copies/ml). As an example, based on population parameter values, the probabilities of detection by nasal and saliva RATs are depicted in S2C Fig.

To account for the practical performance of RATs, we introduce a scaling factor α∈[0,1], reflecting the assay’s sensitivity. The probability of detection by RATs can then be defined as $\alpha \cdot p_{d}(t)$. This parameter allows us to scale down the theoretical detection probability derived from estimated viral dynamics and measurement error, thereby incorporating the reduced sensitivity observed in real-world RAT performance, compared to RT-PCR testing [53–55]. Note that α=0 corresponds to a scenario where no testing is performed.

### Transmissibility after screening with RATs in pre-symptomatic phase

During the pre-symptomatic phase, we considered a one-off screening using a single RAT to detect SARS-CoV-2 infected individuals. The screening period was assumed to be equal to the (mean) serial interval of COVID-19, reflecting a scenario where an infected individual (infectee) suspects infection and seeks testing after recognizing symptoms of a proximate person (infector) (e.g., household or workplace). We also assumed that once an infected individual identifies their infection using the RAT, transmission will be fully prevented due to self-quarantine. As a baseline setting, a single RAT is available within the screening period. Then, the expected number of secondary cases during the pre-symptomatic phase of an infected individual i under screening with a single RAT, $\overset{pre}{\underset{i}{\overset{―}{R}}}$, is defined by


$$\begin{array}{c} \overset{pre}{\underset{i}{\overset{―}{R}}}=\frac{\text{1}}{T_{SP}}\sum_{t=t_{s,i}-T_{SP}}^{t_{s,i}-1}\left[\underset{\text{Detected\textbackslash{} after\textbackslash{} infection}}{\underbrace{p_{d,i}\left(t\right)\overset{t}{\underset{\text{0}}{\int }}\beta_{i}\left(\tau \right)d\tau }}+\underset{\text{Not\textbackslash{} detected\textbackslash{} after\textbackslash{} infection}}{\underbrace{\left(1-p_{d,i}\left(t\right)\right)\overset{t_{s,i}}{\underset{\text{0}}{\int }}\beta_{i}\left(\tau \right)d\tau }}\right], \end{array}$$ (11)

where $T_{SP}$ is the screening period, $t_{s,i}$ is the symptom-onset time for an infected individual i, and $p_{d,i}(t)$ is the probability that the infected individual i is detected by a RAT conducted at time since infection t (where $p_{d}(t)=0$ for t<0, i.e., detection is not possible if the test is conducted prior to infection). Pre-symptomatic screening is evaluated on days $t\in \{t_{s,i}-T_{SP},\cdots ,t_{s,i}-1\}$, i.e., from $T_{SP}$ days before symptom onset up to the day immediately preceding onset). The first term in the summation gives the total infectiousness by time t if the infection is detected at time t. If detection does not occur, then there is no reduction in pre-symptomatic transmission, as represented by the second term. Importantly, $\overset{pre}{\underset{i}{\overset{―}{R}}}$ is computed by uniformly averaging over all possible testing days within the screening window, which reflects variation in symptom recognition and test-access timing. Then, a reduction of transmissibility and a risk of transmission in the pre-symptomatic phase were calculated as $(\overset{pre}{\underset{i}{R}}-\overset{pre}{\underset{i}{\overset{―}{R}}})/\overset{pre}{\underset{i}{R}}\;$ and $\overset{pre}{\underset{i}{\overset{―}{r}}}=1-exp(-\overset{pre}{\underset{i}{\overset{―}{R}}})$, respectively. We set the serial interval to be 6 days as a baseline [56] (i.e., $T_{SP}=6$), and a sensitivity analysis was conducted (from 2 to 7 days) [57].

### Transmissibility after ending isolation with RATs in post-symptomatic phase

During the post-symptomatic phase, we considered transmission after ending isolation with RATs. Supposing that a SARS-CoV-2 infected individual conducts daily RATs starting one day after symptom onset, we assumed that isolation ends when either two consecutive negative test results occur or a maximum isolation period of $T_{IP}=5$ days is reached, aligning with a previous US guideline for COVID-19 patients [29]. Then, the expected number of secondary cases after ending isolation with RATs during the symptomatic phase for infected individual i,$\overset{post}{\underset{i}{\overset{―}{R}}}$, can be obtained as follows:


$$\overset{\text{post}}{\underset{i}{\bar{R}}}=\underset{\text{Ending isolation before full isolation period}}{\underbrace{\sum_{x=2}^{T_{IP}-1}\left(P_{x,i}\int_{t_{s,i}+x}^{\infty }\beta_{i}(\tau )\;d\tau \right)}}+\underset{\text{Full isolation period}}{\underbrace{\left(1-\sum_{x=2}^{T_{IP}-1}P_{x,i}\right)\int_{t_{s,i}+T_{IP}}^{\infty }\beta_{i}(\tau )\;d\tau }},$$ (12)

where the coefficient $P_{x,i}$ corresponds to the probability that the individual i first receives two consecutive negative test results $2\leq x\leq \left(T_{IP}-1\right)$ days since symptom onset (see below). The first term in Eq. (12) represents the total infectiousness when ending isolation x days after symptom onset through two consecutive negative test results. On the other hand, the second term in Eq. (12) means the total infectiousness after the full isolation period, if two consecutive negative test results are not obtained before completing the full isolation period. The probabilities, $P_{x,i}$, of first receiving two consecutive negative test results 2≤x≤4 days after symptom onset (under daily testing, starting one day after symptom onset), are given by


$$P_{2,i}=\left(1-p_{d,i}\left(t_{s,i}+1\right)\right)\left(1-p_{d,i}\left(t_{s,i}+2\right)\right),$$

$$P_{3,i}=p_{d,i}\left(t_{s,i}+1\right)\left(1-p_{d,i}\left(t_{s,i}+2\right)\right)\left(1-p_{d,i}\left(t_{s,i}+3\right)\right),$$

$$P_{4,i}=p_{d,i}\left(t_{s,i}+2\right)\left(1-p_{d,i}\left(t_{s,i}+3\right)\right)\left(1-p_{d,i}\left(t_{s,i}+4\right)\right).$$

Since we took $T_{IP}=5$ days in our main analysis, the above equations were sufficient to analyze the potential ending of isolation before the maximum period. However, in general, for x≥5 days we have


$$P_{x,i}=\left(1-\sum_{y=2}^{x-3}P_{y,i}\right)p_{d,i}\left(t_{s,i}+x-2\right)\left(1-p_{d,i}\left(t_{s,i}+x-1\right)\right)\left(1-p_{d,i}\left(t_{s,i}+x\right)\right).$$

A reduction of transmissibility and a risk of transmission in the post-symptomatic phase were then computed as $(\overset{post}{\underset{i}{R}}-\overset{post}{\underset{i}{\overset{―}{R}}})/\overset{post}{\underset{i}{R}}$ and $\overset{post}{\underset{i}{\overset{―}{r}}}=1-exp(-\overset{post}{\underset{i}{\overset{―}{R}}})$, respectively. In addition, under ending isolation with RATs, we computed the expected isolation period for each individual i as follows:


$$\begin{array}{c} {\overset{―}{IP}}_{i}=\sum_{x=2}^{T_{IP}-1}\left(P_{x,i}\times x\right)+(1-\sum_{x=2}^{T_{IP}-1}P_{x,i})\times T_{IP}. \end{array}$$ (13)

Here, the full isolation period was set to be 5 days as a baseline [29] (i.e., $T_{IP}=5$), and we performed a sensitivity analysis (from 5 to 10 days).

## Results

**Fig 1:**
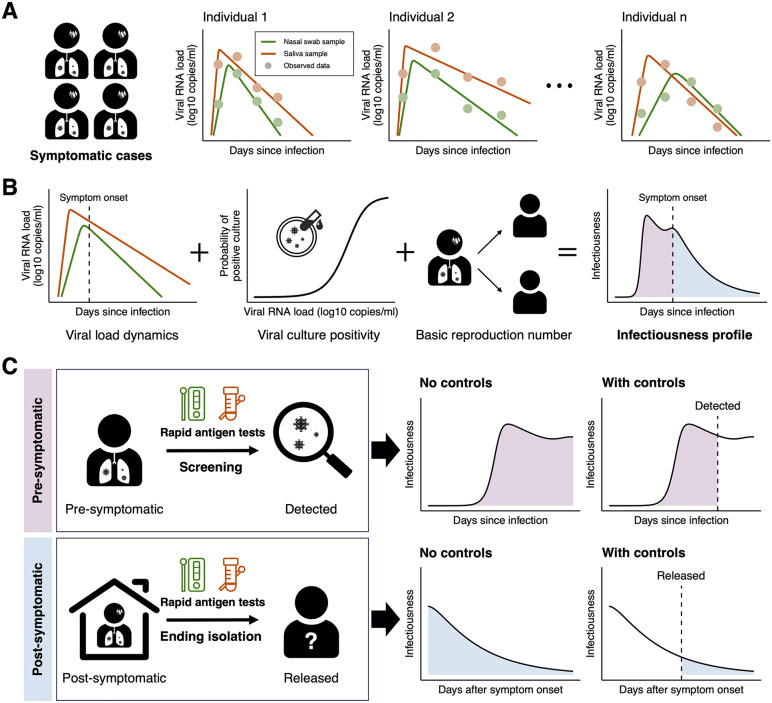
Workflow for analyzing the effectiveness of various RATs at reducing virus transmission in the pre-symptomatic and post-symptomatic phases in different scenarios: (A) A within-host viral dynamics model is fitted to longitudinal viral load data in paired samples (nasal swab and saliva samples) for symptomatic cases, accounting for variability in viral dynamics between individuals. **(B)** Reconstructed viral load dynamics, viral culture positivity, and the basic reproduction number are used to characterize an individual infectiousness profile, over both the pre-symptomatic and post-symptomatic phases. **(C)** The effectiveness of controls can be assessed in each phase: we considered screening with RATs and ending isolation with RATs in the pre-symptomatic and post-symptomatic phases, respectively. Schematic elements and icons in this figure were adapted from open-source resources obtained from Openclipart and Wikimedia Commons. Openclipart resources are in the public domain; Wikimedia Commons materials were used in accordance with their respective Creative Commons licenses.

We aimed to assess the comparative effectiveness of various RATs, as exemplified by nasal and saliva RATs, in mitigating the transmission of respiratory infectious diseases in preparation for the next epidemic or pandemic, as illustrated in Fig 1. As a case study for testing our methods, we considered symptomatic SARS-CoV-2 infections due to the substantial amount of data that are now available. Initially, we characterized the viral dynamics of SARS-CoV-2 infections. A within-host model was fitted to paired nasal swab and saliva samples for symptomatic cases, accounting for variability in viral dynamics between individuals (Fig 1A). To construct an individual infectiousness profile, reconstructed viral load dynamics, viral culture positivity, and the basic reproduction number were used (see **Materials and Methods**), enabling us to characterize “transmissibility” in different phases of a symptomatic infection — specifically, the pre-symptomatic and post-symptomatic phases (Fig 1B). Then, the effectiveness of controls with RATs was assessed in each phase — nasal and saliva RATs were each considered. In the pre-symptomatic phase, we assumed that screening with RATs is used to detect infected individuals, with isolation occurring upon detection (upper panel in Fig 1C). After infected individuals have shown symptoms, we assumed that RATs are used to inform the timing of release from isolation (lower panel in Fig 1C). Consequently, compared to a scenario with no controls, the effectiveness of control measures using different RATs (nasal swab and saliva samples) for reducing transmissibility was analyzed in the pre-symptomatic and post-symptomatic phases, respectively (Fig 1C). While we here focus on clinical and experimental data obtained in the context of COVID-19 patients’ nasal and saliva RATs, we emphasize that our approach can be extended to consider various tests (including more than two different types of test) and to identify the most suitable test for a specific purpose (e.g., to identify pre-symptomatic cases or to determine when individuals can cease isolation). Moreover, our general approach could be applied for a range of different viral pathogens.

### Characterization of SARS-CoV-2 viral dynamics and infectiousness

**Fig 2:**
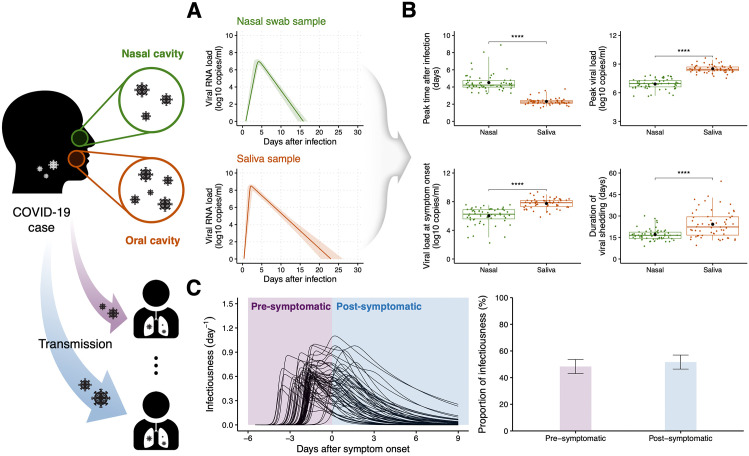
Characterization of SARS-CoV-2 viral dynamics and infectiousness. **(A)** Viral dynamics in the nasal cavity and the oral cavity. The solid lines are the estimated viral load trajectories for a COVID-19 case with paired nasal swab (upper panel) and saliva samples (lower panel) under the best-fitting population parameters. The shaded regions indicate 95% confidence intervals computed using a bootstrap approach. **(B)** Comparison of viral characteristics between nasal swab and saliva samples: peak time, peak viral load, viral load at symptom onset, and duration of viral shedding. The box-and-whisker plots show the medians (50^th^ percentile; bold lines), interquartile ranges (25^th^ and 75^th^ percentiles; boxes), and 2.5^th^ to 97.5^th^ percentile ranges (whiskers). The black dots indicate mean values. The two-sided Mann-Whitney test was used to test for significant differences between the two sample types (ns, p≥0.05; *, p<0.05; **, p<0.01; ***, p<0.001; ****, p<0.0001). **(C)** Estimated individual infectiousness. The black solid lines in the left panel represent individual infectiousness profiles under the best-fitting individual parameters. The purple and blue shaded regions indicate the pre-symptomatic and post-symptomatic phases, respectively. The purple and blue bars in the right panel correspond to mean proportions of infectiousness in the pre-symptomatic and post-symptomatic phases, respectively. The error bars indicate 95% prediction intervals. Schematic elements and icons in this figure were adapted from open-source resources obtained from Openclipart and Wikimedia Commons. Openclipart resources are in the public domain; Wikimedia Commons materials were used in accordance with their respective Creative Commons licenses.

We conducted a literature search for individual-level COVID-19 patient data. We identified a total of 58 symptomatic cases with paired nasal swab and saliva samples meeting our inclusion criteria (see **Materials and Methods** and S1 Table). We then fitted a within-host viral dynamics model to the longitudinal viral load data from the paired samples (Figs 2A and S3). All estimated parameter values are given in S2 Table. To compare the viral dynamics between the two sample types, statistical tests were performed (Fig 2B). The peak viral load time in nasal swab samples was significantly later than in saliva samples (p<0.0001 from the two-sided Mann-Whitney test): 4.5 days (95% CI: 3.3−7.5) and 2.3 days (95% CI: 1.7−3.5) for nasal and saliva samples, respectively. Furthermore, the nasal swab samples had significantly lower peak viral loads than the saliva samples (p<0.0001 from the two-sided Mann-Whitney test): 6.9 log10 copies/ml (95% CI: 5.7−7.5) and 8.5 log10 copies/ml (95% CI: 7.8−9.4) for nasal swab and saliva samples, respectively. There was also a significant difference between viral loads in nasal swab and saliva samples at symptom onset (p<0.0001 from the two-sided Mann-Whitney test): 6.0 log10 copies/ml (95% CI: 3.0−7.5) and 7.7 log10 copies/ml (95% CI: 6.4−9.0) for nasal swab and saliva samples, respectively. Viral shedding was significantly longer in saliva samples (p<0.0001 from the two-sided Mann-Whitney test): 17.0 days (95% CI: 12.1−27.6) and 24.2 days (95% CI: 13.0−42.9) for nasal swab and saliva samples, respectively. These results indicate that the viral dynamics may be different in different organs as reported in previous studies [58–60]. This suggests that utilizing different specimen types may yield different outcomes and have differing implications for the potential to control outbreaks.

To characterize individual infectiousness profiles for viral transmission, we first employed a model describing the probability of transmission [38]. The probabilistic model describes the link between viral dynamics and infectiousness. Infection is initiated by infectious viruses, but viral loads quantified using RT-PCR include both infectious and non-infectious viruses; therefore, viral cell culture positivity data can be used to quantify infectious virus titers. We then obtained an overall transmission probability (i.e., p(t) as described in Eq. (4)) that incorporates viral dynamics from both nasal swab and saliva samples, since the transmission of respiratory viruses is generally associated with droplets and aerosols from the nasal and oral cavities [41]. More detailed derivations are presented in **Materials and Methods** section. In Fig 2C, assuming a basic reproduction number of $R_{\text{0}}=3.0$ [61–63], we characterized the individual infectiousness profiles throughout infection (i.e., β(t) as described in Eq. (6)) for the 58 symptomatic cases using the estimated individual parameters in the viral dynamics model (S3 Fig) and experimental data on the probability of positive culture [39] (S1 Fig) ($R_{\text{0}}$ was varied between 1.5 to 6.0). Due to the variability in viral dynamics between different infected individuals, heterogeneous patterns of SARS-CoV-2 transmission were observed. From the estimated viral dynamics of 58 symptomatic cases, the maximum infectiousness was inferred to occur, on average, −1.4 days (95% PI: −3.5 to 0.8) after symptom onset, which is consistent with a previous study [39] (left panel of Fig 2C). Further, approximately half of total infectiousness, 48.1% (95% PI: 43.0 to 53.8), was inferred to occur prior to the onset of symptoms (right panel of Fig 2C). This is consistent with previous findings of substantial pre-symptomatic transmission [17–19,57]. Even without including the impact of case isolation (which likely increases the realized proportion of pre-symptomatic transmission), this proportion of pre-symptomatic transmission for SARS-CoV-2 is higher than estimates from epidemiological studies for other infectious diseases (which include the effects of isolation): below 20% and about 40% for smallpox [64] and pandemic influenza [65], respectively.

### Pre-symptomatic phase: Evaluation of effectiveness of screening with RATs *in silico*

**Fig 3:**
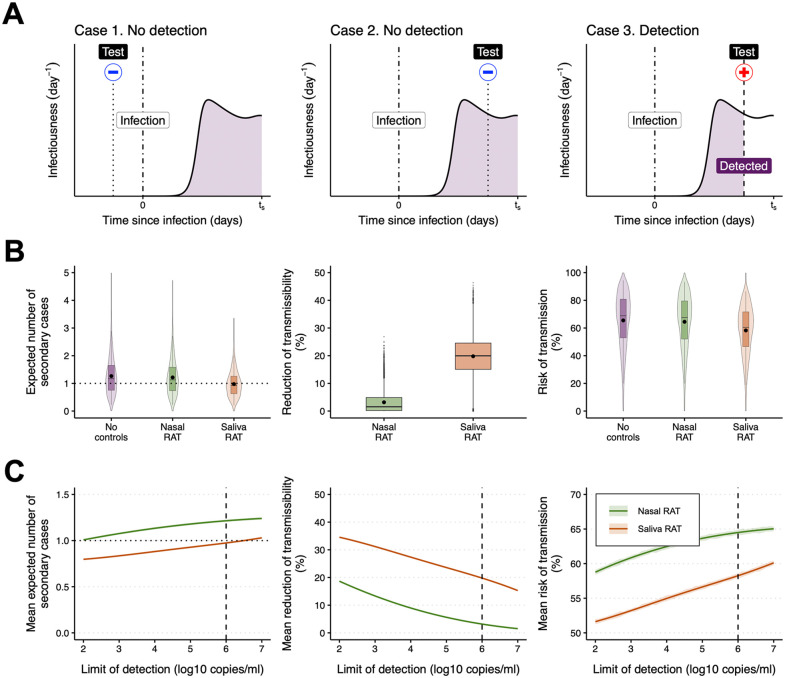
Screening with nasal and saliva RATs in pre-symptomatic phase: (A) Schematic depicting the possible test results when screening with a single RAT in the pre-symptomatic phase. Note that screening began before symptom onset and that a single RAT could be used at a randomly chosen time following a uniform distribution during the screening period. Case 1 represents no detection due to a test conducted before viral infection occurs. Case 2 represents no detection due to a (false) negative test result. Case 3 represents detection due to a positive test result. The times 0 and $t_{s}$ indicate the timing of infection and symptom onset, respectively. The red plus and blue minus signs correspond to positive and negative test results, respectively. **(B)** SARS-CoV-2 transmissibility after screening with one RAT under our baseline assumptions (i.e., screening period = 6 days, $R_{\text{0}}=3.0$, and limit of detection = 6.0 log10 copies/ml). Distribution of the expected number of secondary cases from different individuals without screening ($\overset{pre}{\underset{i}{R}}$) and under screening with either nasal or saliva RATs ($\overset{pre}{\underset{i}{\overset{―}{R}}}$; left panel). The black dots indicate population mean values; $\overset{pre}{\underset{\text{0}}{R}}$ and $\overset{pre}{\underset{\text{0}}{\overset{―}{R}}}$. The horizontal dotted line represents unity. Variation in the reduction of the transmissibility; $(\overset{pre}{\underset{i}{R}}-\overset{pre}{\underset{i}{\overset{―}{R}}})/\overset{pre}{\underset{i}{R}}$ (middle panel). The black dots indicate mean values. Distribution of risk of transmission; $\overset{pre}{\underset{i}{r}}$ and ${\overset{―}{r_{i}}}^{pre}$ (right panel). The black dots indicate mean values; $r^{pre}$ and ${\overset{―}{r}}^{pre}$. The violin plots show the kernel probability density. The box-and-whisker plots show the medians (50^th^ percentile; bold lines), interquartile ranges (25^th^ and 75^th^ percentiles; boxes), and 2.5^th^ to 97.5^th^ percentile ranges (whiskers). **(C)** COVID-19 transmissibility after screening with one RAT for different limits of detection. Population mean number of secondary cases; $\overset{pre}{\underset{\text{0}}{\overset{―}{R}}}$ (left panel). The horizontal dotted line indicates unity. Mean reduction in transmissibility; $\frac{\text{1}}{N}\sum_{i=1}^{N}(\overset{pre}{\underset{i}{R}}-\overset{pre}{\underset{i}{\overset{―}{R}}})/\overset{pre}{\underset{i}{R}}$ (middle panel). Mean risk of transmission; ${\overset{―}{r}}^{pre}$ (right panel). The shaded regions correspond to 95% confidence intervals computed using a bootstrap approach. The vertical dashed lines indicate the baseline limit of detection (6.0 log10 copies/ml).

While RATs represent a useful tool for detecting infections, it can be challenging to detect pre-symptomatic (before symptoms are visible) or asymptomatic infected individuals using RATs. Detection may fail in two scenarios: either the test is conducted before viral infection occurs, or a false negative test result is obtained. Nevertheless, a positive test result enables the identification of infected individuals, aiding in reducing further transmission (Fig 3A). Given that pre-symptomatic infected individuals significantly contribute to transmission for many infectious diseases [66–69], we assessed the impact of screening individuals with saliva-based or nasal-based RATs during the pre-symptomatic phase, as illustrated in Fig 2C. Specifically, we simulated viral dynamics for virtual infected individuals (N=10,000) using our within-host model with estimated parameters for symptomatic COVID-19 (including individual parameter variations), and utilized them for *in silico* analysis. Here, we considered a single RAT available during a 6-day screening period, as assumed to be the mean serial interval of COVID-19 [56]. In other words, it was assumed that screening began 6 days before symptom onset and that a single RAT could be used at a randomly chosen time following a uniform distribution during this period. We also assumed a basic reproduction number, $R_{\text{0}}$, of 3.0 in the absence of testing and a limit of detection (LOD) for RATs of 6.0 log10 copies/ml as baseline settings. Further details on our simulations can be found in the **Materials and Methods** section.

Compared to a scenario with no controls, we analyzed the reduction in transmission during the pre-symptomatic phase that is achieved by screening using nasal and saliva RATs. Without any controls, the expected number of secondary cases generated through pre-symptomatic transmission (i.e., Eq. (7)) was estimated to be $\overset{pre}{\underset{\text{0}}{R}}=1.27$ on average. Under single-test screening with a nasal RAT, only 41.0% (95% CI: 40.0−42.0; binomial proportion) of individuals had the expected number of secondary cases, $\overset{pre}{\underset{i}{\overset{―}{R}}}$, below one, and the mean, $\overset{pre}{\underset{\text{0}}{\overset{―}{R}}}$, was not significantly below one (p≥0.05 from the one-sided t test). By contrast, with saliva RAT screening, 55.8% (95% CI: 54.8−56.8; binomial proportion) had the expected number of secondary cases below one, and the mean was significantly below one (p<0.0001 from the one-sided t test) (left panel of Fig 3B). Screening with a single nasal RAT reduced the transmissibility during the pre-symptomatic phase by approximately 3.2% on average (95% PI:0−13.9). Conversely, saliva RATs decreased the transmissibility by about 19.8% (95% PI: 3.6−34.5) (middle panel of Fig 3B). The narrower spread and the concentration of low reductions for nasal RATs are associated with slower and lower pre-symptomatic viral load in nasal swab samples (Fig 2B) and with the greater difficulty of pre-symptomatic detection (S2C Fig), whereas saliva RATs show a broader distribution because earlier detectability and heterogeneous early viral load trajectories yield a mix of early detections (large reduction) and misses (small reduction) (Fig 2B). The mean risk of (at least one) pre-symptomatic transmission occurring without any controls (i.e., Eq. (9)) was estimated to be $r^{pre}=65.5\%$ (95% PI: 19.9−94.5). Under screening with a single nasal RAT, the mean risk of transmission was estimated to be ${\overset{―}{r}}^{pre}=64.5\%$ (95% PI: 19.9−93.4), whereas screening with a single saliva RAT significantly yielded a lower value of 58.3% (95% PI: 18.7−86.6). There was a statistically significant difference in the mean risk among the three conditions (no controls, nasal RAT, and saliva RAT) (F=418.2,  p<0.0001 from the one-way ANOVA test). Moreover, in 98.9% (95% CI: 98.7−99.1; binomial proportion) of individuals, saliva RATs produced a lower risk, and on average reduced risk more than nasal RATs (p<0.0001 from the one-sided t test) (right panel of Fig 3B). In a sensitivity analysis allowing overdispersion via a negative binomial offspring distribution (see **Materials and Methods**), the mean risk of pre-symptomatic transmission (≥1 secondary case) decreased compared with the Poisson baseline, whereas the mean risk for higher-count events (e.g., ≥4 secondary cases) increased. Importantly, the strategy ranking was unchanged; saliva RAT still yielded lower transmission risk than nasal RAT (p<0.0001 from the one-sided t test) (S4A Fig).

Even when using nasal RATs with a lower LOD (i.e., high sensitivity RATs) compared to the baseline, the average number of secondary cases after screening with RATs, $\overset{pre}{\underset{\text{0}}{\overset{―}{R}}}$, remained greater than one: 1.21 (95% CI: 1.20−1.23) (left panel of Fig 3C). The mean reduction in transmission remained approximately 17% lower for nasal than saliva RATs, regardless of the LOD (middle panel of Fig 3C). In addition, under screening with a high-sensitivity nasal RAT (e.g., LOD = 2.0 log10 copies/ml), the mean risk of transmission, ${\overset{―}{r}}^{pre}$, was 58.8% (95% CI: 58.4−59.1), whereas a saliva RAT reduced it to 51.6% (95% CI: 51.3−51.9), a significantly lower value (p<0.0001 from the one-sided t test) (right panel of Fig 3C). We also reported the absolute difference in the mean risk of transmission between nasal and saliva RATs. At the baseline setting, the mean risk difference was 6.2% (95% CI: 6.1−6.3). As sensitivity increased (lower LOD), the difference rose slightly relative to the baseline; across the LOD range, the mean remaining risk with nasal RATs was significantly higher than that with saliva RATs (p<0.0001 from the one-sided t test) (left panel of S5 Fig). This pattern likely reflects the greater advantage of saliva sampling for early detection (Fig 2A and 2B). Although comparing the accuracy of these two samples has been a subject of debate [8,9,55,70,71], our results suggest that saliva RATs may prevent transmission from pre-symptomatic individuals more effectively than nasal RATs.

Additionally, we conducted sensitivity analyses to assess the impact of different factors. First, we varied the screening period in the pre-symptomatic phase. As the screening period was extended, that is, screening was initiated earlier, the mean risk of transmission increased, since testing was then more likely to take place before or shortly after infection (when a negative test result is likely). However, again, with a nasal RAT, the mean risk of transmission was substantially higher than with a saliva RAT (S6A Fig). Second, we considered different values of the basic reproduction number, $R_{\text{0}}$ (see S3 Table). As the value of $R_{\text{0}}$ increased, a substantial increase in infectiousness was observed around symptom onset, leading to high transmissibility during the pre-symptomatic phase (S7A Fig). The mean risk of transmission for both nasal and saliva RATs also increased, while the mean risk difference decreased – to 4.2% (95% CI: 4.1−4.2) at $R_{\text{0}}=6.0$. When $R_{\text{0}}\leq 1.8$, the mean risk of transmission during the pre-symptomatic phase was below 50% with a nasal RAT. However, it remained about 6% higher than the corresponding value with a saliva RAT (p<0.0001 from the one-sided t test) (left panel of S8 and S7B Figs).

However, in real-world settings, the sensitivity of testing can vary depending on the type of sample and other external factors. Thus, to account for the practical performance of RATs, we introduced a scaling factor α∈[0,1] applied to the probability of detection, reflecting the assay’s sensitivity (see **Materials and Methods**). Overall, the mean risk of transmission slightly declined as α increased, indicating that higher test sensitivity contributes to better transmission control. However, the nasal RAT had a rather limited impact on reducing transmission in this phase. Interestingly, even with lower α (lower sensitivity), the nasal RAT reduced the risk of transmission less than the saliva RAT across the entire range of α (S9A Fig). This implies that, despite its lower analytical sensitivity in real-world settings, the saliva RAT might be more effective than the nasal RAT in screening during the pre-symptomatic phase – possibly due to earlier or higher detectability of viral load in saliva samples (Fig 2A and 2B).

### Post-symptomatic phase: Evaluation of effectiveness of ending isolation with RATs *in silico*

**Fig 4:**
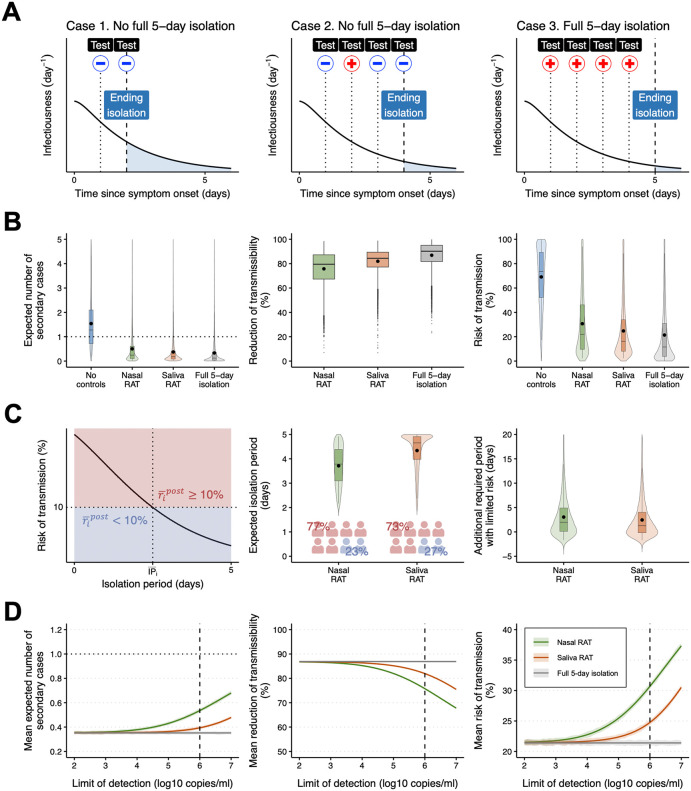
Ending isolation based on consecutive negative test results from nasal and saliva RATs in the post-symptomatic phase: (A) Schematic illustrating the use of RATs to determine when isolation can end. Case 1 represents ending isolation due to two consecutive negative test results one and two days after starting isolation. Case 2 represents another scenario of ending isolation due to two consecutive negative test results before the full 5-day isolation period. Case 3 represents ending isolation after the full 5-day isolation period since two consecutive negative test results were not obtained. The red plus and blue minus signs correspond to positive and negative test results, respectively. **(B)** Transmissibility after ending isolation with RATs under our baseline parameter values for COVID-19 (maximum isolation period =5 days, $R_{\text{0}}=3$, and limit of detection =6.0 log10 copies/ml). Distribution of the expected number of secondary cases by different individuals without any controls ($\overset{post}{\underset{i}{R}}$) and under different isolation strategies ($\overset{post}{\underset{i}{\overset{―}{R}}}$; left panel). The black dots indicate population mean values; $\overset{post}{\underset{\text{0}}{R}}$ and $\overset{post}{\underset{\text{0}}{\overset{―}{R}}}$. The horizontal dotted line represents unity. Distribution of the reduction in transmissibility; $(\overset{post}{\underset{i}{R}}-\overset{post}{\underset{i}{\overset{―}{R}}})/\overset{post}{\underset{i}{R}}$(middle panel). The black dots indicate mean values. Distribution of the risk of transmission; $\overset{post}{\underset{i}{r}}$ and ${\overset{―}{r_{i}}}^{post}$(right panel). The black dots indicate mean values; $r^{post}$ and ${\overset{―}{r}}^{post}$. **(C)** Evaluation of isolation periods with RATs under our baseline parameter values. Schematic depicting an individual’s risk of transmission after different possible isolation periods (left panel). The red-colored and blue-colored regions correspond to a risk of transmission after ending isolation (i.e.,${\overset{―}{r_{i}}}^{post}$) above and below10%, respectively. Note that those two regions differ between individuals. Distribution of the mean isolation period following symptom onset, ${\overset{―}{IP}}_{i}$(middle panel). Distribution of the additional required period with limited risk (right panel). Note that a positive value represents the additional period of isolation (after the isolation period determined using RATs) required to lower an individual’s risk of transmission below 10%. A negative value characterizes an unnecessary portion of the isolation period for an individual whose risk of transmission after ending isolation with RATs is below 10%. The violin plots show the kernel probability density. The box-and-whisker plots show the medians (50^th^ percentile; bold lines), interquartile ranges (25^th^ and 75^th^ percentiles; boxes), and 2.5^th^ to 97.5^th^ percentile ranges (whiskers). **(D)** Transmissibility after ending isolation with RATs for different limits of detection. Population mean number of secondary cases; $\overset{post}{\underset{\text{0}}{\overset{―}{R}}}$(left panel). The horizontal dotted line indicates unity. Mean reduction of transmissibility; $\frac{\text{1}}{N}\sum_{i=1}^{N}(\overset{post}{\underset{i}{R}}-\overset{post}{\underset{i}{\overset{―}{R}}})/\overset{post}{\underset{i}{R}}$ (middle panel). Mean risk of transmission; ${\overset{―}{r}}^{post}$(right panel). The shaded regions correspond to 95% confidence intervals computed using a bootstrap approach. The vertical dashed lines indicate the baseline limit of detection (6.0 log10 copies/ml).

Another important application of RATs for guiding NPIs is determining the appropriate time at which individuals are permitted to end isolation. Specifically, shortening the isolation period while maintaining an acceptably small risk of additional transmission occurring is useful for reducing costs associated with isolation [24,25]. A range of different isolation periods were recommended in different countries at different stages of the COVID-19 pandemic. For example, in the later part of the acute phase of the pandemic in countries such as the UK and the USA, a 5-day isolation period was recommended for COVID-19 patients exhibiting symptoms [29], which was shorter than the isolation periods recommended earlier in the pandemic. Policies were also introduced under which RATs could be employed to determine when isolation can be ended safely, for example, with the requirement of two consecutive negative test results prior to the end of isolation [29]. In our simulations, we considered a similar criterion under the same baseline settings (i.e., $R_{\text{0}}=3.0$ and a LOD of 6.0 log10 copies/ml) with daily RATs following symptom onset: if two consecutive negative test results are obtained before the completion of 5 days isolation period, isolation could be discontinued. However, COVID-19 patients must continue with the full isolation protocol if positive test results persist (Fig 4A).

The expected number of secondary cases during the post-symptomatic phase (i.e., Eq. (7)) was $\overset{post}{\underset{\text{0}}{R}}=1.73$ on average if no controls were implemented (i.e., symptomatic individuals were not isolated). However, a full 5-day isolation period substantially reduced the expected number of secondary cases after isolation. Moreover, if either type of RAT was used to facilitate early release from isolation (following two consecutive negative tests), the post-isolation reproduction number (i.e., $\overset{post}{\underset{\text{0}}{\overset{―}{R}}}$ as described in Eq. (12)) decreased: 85.7% (95% CI: 85.0−86.3; binomial proportion) and 91.2% (95% CI: 90.6−91.7; binomial proportion) of individuals had $\overset{post}{\underset{i}{\overset{―}{R}}}\leq 1$ with nasal and saliva RATs, respectively, and the mean, $\overset{post}{\underset{\text{0}}{\overset{―}{R}}}$, was significantly below one in both cases (p<0.0001 from the one-sided t test) (left panel of Fig 4B). Ending isolation with RATs reduced average transmissibility during the post-symptomatic phase by 75.8%(95% PI: 39.6−94.6) and 82.0%(95% PI: 54.5−95.3) for nasal and saliva RATs, respectively (middle panel of Fig 4B), compared to the no-control scenario. The mean risk of post-symptomatic transmission without any controls (i.e., $r^{post}$ as described in Eq. (10)) was estimated to be 69.1%(95% PI: 17.2−99.7). When isolation was ended based on two consecutive negative test results, the risk of transmission, ${\overset{―}{r}}^{post}$, was below 50% on average: 30.7% (95% PI: 2.3−94.0) with nasal RATs and 24.8% (95% PI: 2.3−88.5) with saliva RATs. Across the three conditions – no controls, nasal RAT, and saliva RAT – the mean risks differed significantly (F=8348,  p<0.0001 from the one-way ANOVA test). In addition, 75.6% (95% CI: 74.8−76.5; binomial proportion) of individuals showed lower risk with saliva RATs, and the risk was significantly more reduced than with nasal RATs (p<0.0001 from the one-sided t test). For comparison, the full 5-day isolation reduced the risk to 21.4% (95% PI: 0.3−88.4) (right panel of Fig 4B). Taken together, these results suggest that saliva RATs may be more effective at reducing transmission than nasal RATs in the context of determining the end of isolation. In the post-symptomatic phase, allowing overdispersion likewise lowered the mean risk of transmission (≥1 secondary case) compared with the Poisson baseline, while increasing the risk of higher-count events (≥4 secondary cases). Again, the strategy ranking remained unchanged, with saliva RATs yielding lower transmission risk than nasal RATs (p<0.0001 from the one-sided t test) (S4B Fig).

We also analyzed the time of ending isolation with RATs under our baseline parameter values. Specifically, we computed the mean isolation period for each individual (i.e., ${\overset{―}{IP}}_{i}$ as described in Eq. (13)) and examined whether the individual’s risk of transmission after ending isolation, ${\overset{―}{r_{i}}}^{post}$, remained above 10% (left panel of Fig 4C). The mean isolation period was estimated to be 3.7 days (95% PI: 2.2−4.9) and 4.3 days (95% PI: 2.4−5.0) using nasal and saliva RATs, respectively. Since lower viral loads and faster viral clearance were observed in the nasal cavity than in the oral cavity (Fig 2B), nasal RATs were more likely to lead to negative test results occurring earlier, allowing isolation to end sooner. However, if nasal RATs were used, about 77% of all individuals ended isolation, with their risk of transmission still being above10%. On the other hand, about 73% of infected individuals had a risk of transmission higher than 10% after ending isolation when saliva RATs were used to determine the end of isolation (middle panel of Fig 4C). In addition, for each individual, we computed the additional period of isolation that would be required to reduce the individual’s risk of post-isolation transmission to below 10% (termed the “additional required period with limited risk”). Specifically, we identified the earliest day at which the risk of post-isolation transmission fell below 10%, $\overset{*}{\underset{i}{d}},$ and defined the additional required isolation as $\Delta_{i}=\overset{*}{\underset{i}{d}}-{\overset{―}{IP}}_{i}$. Positive values ($\Delta_{i}>0$) indicate that extra days are needed, whereas negative values ($\Delta_{i}<0$) indicate that the risk was already below 10% before ${\overset{―}{IP}}_{i}$ (i.e., potentially unnecessary isolation). We found that the population average additional required periods to limit the risk to 10% were 3.3 days (95% PI: −1.7 to 16.1) and 2.7 days (95% PI: −1.7 to 15.0) for nasal and saliva RATs, respectively. This suggests that both types of RATs could be equally effective when considering the estimated additional isolation after the expected isolation period for each test – the risk of transmission remains below 10%, with an average total isolation period of about 7 days (middle and right panels of Fig 4C).

Additionally, we analyzed transmissibility during the post-symptomatic phase under different LODs. Under the criterion of requiring two consecutive negative test results to end isolation, the population mean number of secondary cases after ending isolation, $\overset{post}{\underset{\text{0}}{\overset{―}{R}}}$, with nasal and saliva RATs, was below one for every LOD considered (left panel of Fig 4D). When high-sensitivity RATs (e.g., LOD = 2.0 log10 copies/ml) were used, the mean risk of post-isolation transmission, ${\overset{―}{r}}^{post}$, for either nasal or saliva RATs was comparable to the approximately 20% for the full 5-day isolation period: 21.5% (95% CI: 21.1−22.0) with nasal RATs and 21.4% (95% CI: 21.0−21.9) with saliva RATs. This is because negative test results before completing 5 days of isolation are unlikely with much higher-sensitivity RATs (right panel of Fig 4D). Thus, as sensitivity increased (lower LOD), the mean risk difference approached zero; under the baseline setting, however, it was 5.9% (95% CI: 5.7−6.1), indicating that the risk with nasal RATs was significantly higher than that with saliva RATs (p<0.0001 from the one-sided t test) (right panel of S5A Fig).

We also performed sensitivity analyses in which we varied the full isolation period ($T_{IP}$) and the basic reproduction number ($R_{\text{0}}$). For all cases with a full isolation period longer than 5 days, the mean risk of post-isolation transmission using RATs was below 50% (S6B Fig). Even if the full isolation period increased, using low-sensitivity RATs resulted in a shortened isolation period, relative to the corresponding full isolation period (S10 Fig). However, in such cases there may be a high risk of further transmission after ending isolation (right panel of Fig 4D). In addition, under a full isolation period of 5 days and a LOD of 6.0 log10 copies/ml, ending isolation with nasal and saliva RATs could reduce the mean risk of post-symptomatic transmission below 50% even at higher $R_{\text{0}}$ values (S7C Fig). The mean risk difference also increased slightly with $R_{\text{0}}$, reaching 7.0% (95% CI: 6.8−7.2) at $R_{\text{0}}=6.0$ (right panel of S8 Fig). As in the pre-symptomatic phase, the effectiveness in reducing transmissibility during the post-symptomatic phase was lower for nasal than for saliva RATs; however, in this phase, the effect of isolation itself on preventing further transmission was notable. These analyses demonstrate how parameter values in our approach can be changed to reflect situation-specific factors, enabling its application for different viral pathogens.

As in the pre-symptomatic phase, we incorporated the scaling factor into the detection probability to account for the practical sensitivity of RATs in the post-symptomatic phase. When the value of α is the same, nasal RATs led to a higher risk of transmission than saliva RATs. However, if the lower α (lower sensitivity) of saliva RATs observed in real-world settings is taken into account [53–55], nasal RATs would likely be more effective at controlling transmissions (S9B Fig). At low α, RATs are more likely to return false-negative results, potentially resulting in premature release from isolation even though infected individuals still have high infectiousness. Consequently, despite the modeled reduction in transmission risk, the practical effectiveness of saliva RATs may be lower than that of nasal RATs, particularly when used to determine the end of isolation under suboptimal sensitivity conditions.

## Discussion

RATs are valuable resources for responding to emerging infectious diseases, enabling infections to be detected quickly and providing a method for determining when individual-specific measures such as isolation can be lifted. However, different types of RAT exist, including those that utilize nasal swab samples and those that utilize saliva samples [5]. Fast identification of which RAT is most effective for the disease and objective under consideration is essential for NPIs to be optimized.

In our recent series of studies on COVID-19, we have investigated the optimization of screening strategies using RATs [72] and have developed guidelines for ending isolation when negative RAT results are obtained [25]. These studies considered RATs based on nasal swab samples, and did not consider saliva-based RATs. Moreover, those previous studies used a single threshold for viral replication based on cell culture experiments to characterize a binary relationship between viral load and infectiousness, whereas in reality the extent of infectiousness (and thus the risk of transmission) depends continuously on viral load. This study comprehensively addresses these issues. Specifically, our computational framework quantifies variations in infectiousness during infection, while also accounting for the numbers of contacts between individuals (Fig 1B). This enabled us to develop more refined isolation guidelines compared to previous studies [24–26]. Furthermore, since our approach is flexible enough to characterize viral dynamics in different sites and accommodate various situation-specific factors, it can be used to evaluate the effectiveness of different RATs in multiple scenarios.

In this study, as an example, we compared the effectiveness of NPIs informed by the results of nasal and saliva RATs for mitigating SARS-CoV-2 transmission in different phases of symptomatic infection. We showed that, if saliva RATs are used to detect infected individuals before symptom onset, then pre-symptomatic transmission could be reduced; our model predicts a larger reduction in pre-symptomatic transmission using saliva RATs than nasal RATs. For individuals who have shown symptoms, a rule involving ending isolation based on consecutive negative results from either nasal or saliva RATs is predicted to be effective at limiting further transmission.

These results suggest that RATs that utilize saliva samples are a useful alternative to those that utilize nasal samples for controlling SARS-CoV-2 transmission, even though nasal RATs are currently the most widely used RAT type [8–13]. The use of saliva RATs may be particularly beneficial in some specific situations given the relative difficulty of using nasal RATs (or other testing types, e.g., PCR tests), for example in elderly care facilities due to the less invasive sampling.

We observed different viral kinetics between nasal and saliva samples in paired sample data. SARS-CoV-2 typically proliferated in the oral cavity rapidly, and the viral load reached its peak before symptoms were present [73], which supports previous evidence that saliva tests may serve as an effective tool for early diagnosis of COVID-19 infection [74]. After symptom onset, viral shedding in the nasal cavity was shorter than in the oral cavity. Therefore, if nasal RATs are utilized, COVID-19 patients are likely to obtain negative results more rapidly, allowing them to end their isolation sooner. However, this may lead to a substantial risk of further transmission due to prematurely ending isolation [25]. Consequently, the use of saliva RATs may be a safer choice for determining when COVID-19 patients can stop isolating. We note that, while saliva RATs may be more effective in reducing SARS-CoV-2 transmission than nasal RATs, the optimal type of test to use may vary for different pathogens and other external factors.

Prior work has also suggested that saliva can enable earlier detection than nasal sampling in acute SARS-CoV-2 infection and has described culture-calibrated viral load kinetics and heterogeneity [35]. Building on that foundation, our study translated these biological differences into policy-relevant performance through in silico experiments. We jointly modeled paired nasal and saliva trajectories, quantified detectability as a time-varying probability, and mapped RNA load to a continuous infectiousness profile calibrated by cell culture data rather than a binary threshold. We then evaluated matched testing protocols in head-to-head simulations for two objectives: screening before symptom onset and ending isolation after symptom onset. This approach moves beyond detectability signals to quantify operational impact, and such purpose-specific intervention comparisons with paired uncertainty have not been presented previously.

The potential applications of our epidemiological modelling framework are wide-ranging. For different pathogens, alternative models describing the link between within-host viral dynamics and between-host transmission could be used. In principle, it would be possible to investigate the effectiveness of different types of RAT for reducing the risk of localized outbreaks [23], or to assess RAT-based screening programs for mitigating transmission within specific establishments (e.g., schools or workplaces) — different RAT types, testing frequencies, and timings of screening initiation could be compared. This would enable us to design situation-specific screening programs, potentially enabling educational and economic activities to be conducted while limiting transmission [22,72].

As with any modelling study, our approach included assumptions and simplifications. First, our results depend on the patient data that we collected, which do not include information regarding various factors (e.g., vaccination history, treatments, past infection, and clinical features). The viral dynamics model was calibrated using data from unvaccinated and untreated patients, but for COVID-19 currently, most infected individuals are likely to have received COVID-19 vaccinations (although, for many individuals, vaccination-induced immunity may have waned [75]). Further investigation into the relationship between those factors and viral load would be required to describe the heterogeneity in infectiousness profiles between infected individuals fully. Second, we only used data from patients who developed symptoms. In our previous study, we found a difference in viral kinetics between symptomatic and asymptomatic individuals, which may lead to different transmissibility between those individuals [25]. Our analysis could be extended to consider individuals with different levels of symptoms if relevant patient data are available. Third, while our data were confined to COVID-19, our approach could be extended and applied to other infectious diseases for which RATs are available, such as influenza and respiratory syncytial virus (RSV). Our framework is pathogen-agnostic in structure – the mapping from the within-host viral load to infectiousness, the testing/decision modules, and the simulation process that generates virtual individuals and summarizes the population-level outcomes. By contrast, we must re-estimate pathogen/test-specific inputs; specimen-specific viral load kinetics, a cell-culture positivity curve linking viral load to infectious virus, assay characteristics by specimen (e.g., limit of detection and sensitivity), and a transmission-scaling parameter calibrated to the target basic reproduction number for the chosen context. Applying the framework to influenza or RSV would therefore amount to refitting these inputs, with full re-calibration/validation beyond SARS-CoV-2 left for future work. Fourth, we assumed homogeneous mixing and did not model age or setting structure, network contacts, or behavioral differences in sampling; such heterogeneity can shift absolute impact [76]. Nonetheless, because our comparison is driven by detection timing by specimen, the relative ranking of nasal- and saliva-based RATs is expected to hold unless subgroup-specific kinetics or field performance by specimen differ substantially; in settings where sampling acceptability matters for repeated testing, saliva is often less invasive and more acceptable and remains a viable alternative in practice [36,77,78]. Fifth, to quantify transmission risk we used a Poisson offspring model for simplicity and interpretability. Because SARS-CoV-2 transmission is overdispersed with documented superspreading, the variance of the offspring distribution often exceeds the mean, leading to a heavy tail [76,79,80]. In our sensitivity analysis using a negative binomial offspring model, the absolute risks shifted; however, the relative ranking between nasal and saliva RATs did not change (S4 Fig). Last, we did not account for alternative factors that may affect antigen test results. In particular, imprecise sampling can occur if RATs are self-administered, particularly for invasive tests that may not be applied correctly. However, in principle, imprecise testing could be incorporated into our probabilistic model describing changes in the probability of test positivity during infection – suggesting that the optimal type of testing may vary depending on sensitivity (S9 Fig). Therefore, when evaluating the practical use of RATs, real-world variabilities in test sensitivity and sample collection should be carefully considered.

Overall, our study provides: (i) a computational approach for linking within-host viral dynamics at different sites to infectiousness; (ii) a framework for evaluating the effectiveness of different RATs for different purposes, and; (iii) potential insights into the effectiveness and uses of saliva samples in the context of COVID-19. We hope that the framework that we have developed will be applied to viruses beyond COVID-19, and thus play a crucial role in improving public health outcomes during future outbreaks of emerging infectious diseases.

## Supporting information

S1 Fig Estimated probability of positive viral culture as a function of SARS-CoV-2 viral RNA load: The solid line corresponds to the estimated trajectory under the best-fitting parameters. Dots indicate reported probabilities of cell culture positivity.
(DOCX)

S2 FigAn illustrative example of infectiousness and probability of detection by rapid antigen tests for a COVID-19 case: (A) SARS-CoV-2 viral load trajectory with paired nasal swab and saliva samples under the best-fitting population parameters.(B) Infectiousness in pre-symptomatic and post-symptomatic phases, based on the estimated SARS-CoV-2 viral load trajectory in (A). (C) Probability of detection by rapid antigen tests under the baseline setting (limit of detection = 6.0 log10 copies/ml), based on the estimated SARS-CoV-2 viral load trajectory in (A). The black vertical dotted lines indicate the timing of symptom onset.(DOCX)

S3 Fig Estimated individual viral load trajectory for each COVID-19 case with paired nasal swab and saliva samples: The solid lines are estimated SARS-CoV-2 viral load trajectories under the best-fitting individual parameters. The shaded regions indicate 95% confidence intervals. The green and red dots represent measured viral load observations. The grey dots correspond to observations where the measured viral load was below the limit of detection.
(DOCX)

S4 FigComparison of risk of transmission under Poisson and negative binomial offspring distributions across control strategies.**(A)** Risk of transmission during the pre-symptomatic phase, defined as the probability that an infected individual generates at least n secondary cases (≥n) over the screening period. **(B)** Risk of transmission during the post-symptomatic phase, defined analogously over the isolation period. For each panel, the x-axis shows the secondary case threshold (n=1 or 4; for n=4, we use 1−F(3), where F is the cumulative distribution function) and the y-axis shows the risk of transmission (%). Columns correspond to scenarios with no controls (left), nasal RAT (middle), and saliva RAT (right). The violin plots show the kernel probability density. The box-and-whisker plots show the medians (50^th^ percentile; bold lines), interquartile ranges (25^th^ and 75^th^ percentiles; boxes), and 2.5^th^ to 97.5^th^ percentile ranges (whiskers). Colored violins indicate the distribution of risks under a Poisson offspring model, and gray violins indicate a negative binomial offspring model with dispersion parameter k=0.41. All values were calculated under the baseline settings (screening period = 6 days, full isolation period = 5 days, limit of detection = 6.0 log10 copies/ml, and basic reproduction number $R_{\text{0}}=3$).(DOCX)

S5 Fig Sensitivity of the difference in mean risk of transmission between saliva and nasal rapid antigen tests to the limit of detection. Left and right panels show the pre-symptomatic and post-symptomatic phases, respectively. The vertical dashed lines indicate the baseline limit of detection (6.0 log10 copies/ml). The shaded regions correspond to 95% confidence intervals computed using a bootstrap approach.
(DOCX)

S6 FigComparison of mean risk of transmission between nasal and saliva rapid antigen tests under different scenarios:(A) Mean risk of transmission after screening with one RAT (i.e., ${\overset{―}{r}}^{pre}$) in the pre-symptomatic phase. The x-axis and y-axis represent the limit of detection and the screening period, respectively. (B) Mean risk of transmission after ending isolation with RATs (i.e., ${\overset{―}{r}}^{post}$) in the post-symptomatic phase. The x-axis and y-axis represent the limit of detection and the full isolation period, respectively. All values were calculated under the baseline value of the basic reproduction number ($R_{\text{0}}=3$). The white color regions indicate a risk of 50%.(DOCX)

S7 FigComparison of risk of transmission between using nasal and saliva rapid antigen tests under different values of basic reproduction number: (A) Estimated mean infectiousness in the absence of controls.The x-axis and y-axis represent days after symptom onset and the value of the basic reproduction number (i.e., $R_{\text{0}}$), respectively. (B) Mean risk of transmission after screening with one RAT (i.e., ${\overset{―}{r}}^{pre}$) in the pre-symptomatic phase. All values were calculated under the baseline settings (screening period = 6 days and limit of detection = 6.0 log10 copies/ml). (C) Mean risk of transmission after ending isolation with RATs (i.e., ${\overset{―}{r}}^{post}$) in the post-symptomatic phase. All values were calculated under the baseline settings (full isolation period = 5 days and limit of detection = 6.0 log10 copies/ml). The shaded regions correspond to 95% confidence intervals computed using a bootstrap approach. The black vertical dashed lines indicate the baseline value of the basic reproduction number ($R_{\text{0}}=3$).(DOCX)

S8 FigSensitivity of the difference in mean risk of transmission between saliva and nasal rapid antigen tests to the basic reproduction number.Left and right panels show the pre-symptomatic and post-symptomatic phases, respectively. The vertical dashed lines indicate the baseline value of the basic reproduction number ($R_{\text{0}}=3$). The shaded regions correspond to 95% confidence intervals computed using a bootstrap approach.(DOCX)

S9 FigComparison of mean risk of transmission between nasal and saliva rapid antigen tests under different values of scaling factor:(A) Mean risk of transmission after screening with one RAT (i.e., ${\overset{―}{r}}^{pre}$) in the pre-symptomatic phase. The values were calculated under the baseline settings (screening period = 6 days and limit of detection = 6.0 log10 copies/ml). **(B)** Mean risk of transmission after ending isolation with RATs (i.e., ${\overset{―}{r}}^{post}$) in the post-symptomatic phase. The values were calculated under the baseline settings (full isolation period = 5 days and limit of detection = 6.0 log10 copies/ml). All values were calculated under the baseline value of the basic reproduction number ($R_{\text{0}}=3$).(DOCX)

S10 FigEvaluation of ending isolation with RATs under different scenarios in the post-symptomatic phase: The expected isolation period under nasal RATs (left panel).The expected isolation period under saliva RATs (right panel). The x-axis and y-axis represent the limit of detection and the full isolation period, respectively. All values were calculated under the baseline value of the basic reproduction number ($R_{\text{0}}=3$).(DOCX)

S1 Table Summary of SARS-CoV-2 viral load data with paired nasal swab and saliva samples. (DOCX)

S2 Table Estimated fixed effect parameters, standard deviation of random effects, and standard deviation of error in SARS-CoV-2 log viral loads from nasal swab and saliva samples. (DOCX)

S3 Table Parameter values for modeling transmissibility of SARS-CoV-2. (DOCX)
