# Supplementary material for: Identifying the optimal rapid antigen test for screening and determining the end of isolation: A modeling study
Source: PLoS Comput Biol. 2026 Apr 2;22(4):e1013102. doi: 10.1371/journal.pcbi.1013102 (PMC13082731; doi:10.1371/journal.pcbi.1013102)
Supplement: S1 Fig — Dots indicate reported probabilities of cell culture positivity. (DOCX) [file pcbi.1013102.s001.docx]

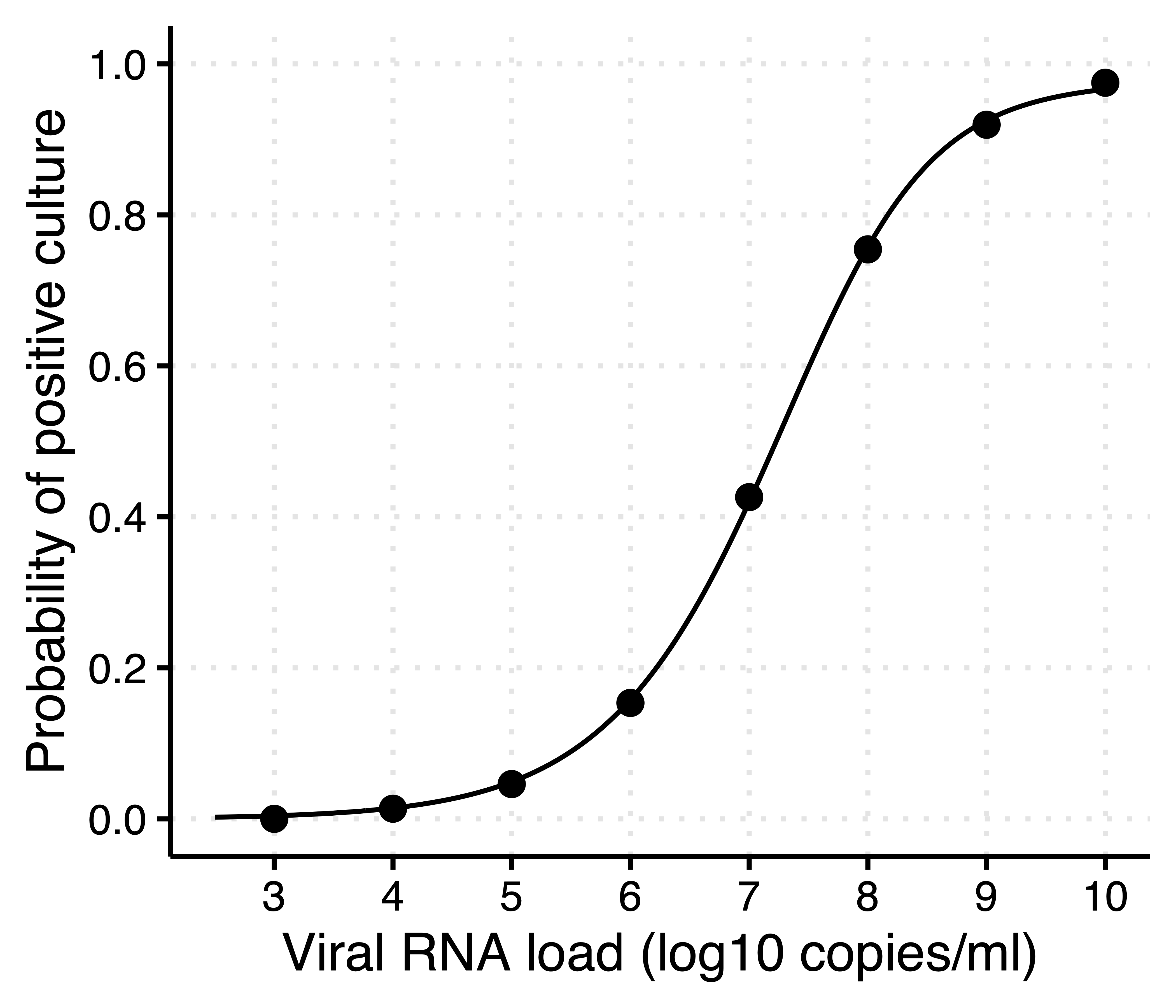


S1 Fig. | Estimated probability of positive viral culture as a function of SARS-CoV-2 viral RNA load: The solid line corresponds to the estimated trajectory under the best-fitting parameters. Dots indicate reported probabilities of cell culture positivity [[1](#_ENREF_1)].
