# Supplementary material for: Identifying the optimal rapid antigen test for screening and determining the end of isolation: A modeling study
Source: PLoS Comput Biol. 2026 Apr 2;22(4):e1013102. doi: 10.1371/journal.pcbi.1013102 (PMC13082731; doi:10.1371/journal.pcbi.1013102)
Supplement: S2 Fig — (B) Infectiousness in pre-symptomatic and post-symptomatic phases, based on the estimated SARS-CoV-2 viral load trajectory in (A). (C) Probability of detection by rapid antigen tests under the baseline setting (limit of detection = 6.0 log10 copies/ml), based on the estimated SARS-CoV-2 viral load trajectory in (A). The black vertical dotted lines indicate the timing of symptom onset. (DOCX) [file pcbi.1013102.s002.docx]

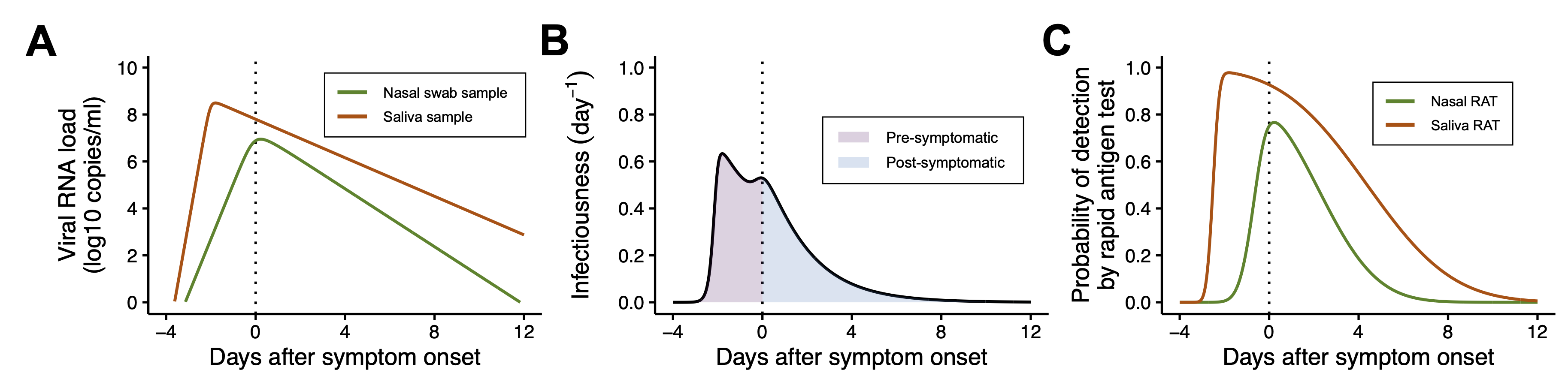


S2 Fig. | An illustrative example of infectiousness and probability of detection by rapid antigen tests for a COVID-19 case: (A) SARS-CoV-2 viral load trajectory with paired nasal swab and saliva samples under the best-fitting population parameters. (B) Infectiousness in pre-symptomatic and post-symptomatic phases, based on the estimated SARS-CoV-2 viral load trajectory in (A). (C) Probability of detection by rapid antigen tests under the baseline setting (limit of detection $\boldsymbol{= 6.0}$ log10 copies/ml), based on the estimated SARS-CoV-2 viral load trajectory in (A). The black vertical dotted lines indicate the timing of symptom onset.
