# Supplementary material for: Identifying the optimal rapid antigen test for screening and determining the end of isolation: A modeling study
Source: PLoS Comput Biol. 2026 Apr 2;22(4):e1013102. doi: 10.1371/journal.pcbi.1013102 (PMC13082731; doi:10.1371/journal.pcbi.1013102)
Supplement: S3 Fig — The shaded regions indicate 95% confidence intervals. The green and red dots represent measured viral load observations. The grey dots correspond to observations where the measured viral load was below the limit of detection. (DOCX) [file pcbi.1013102.s003.docx]

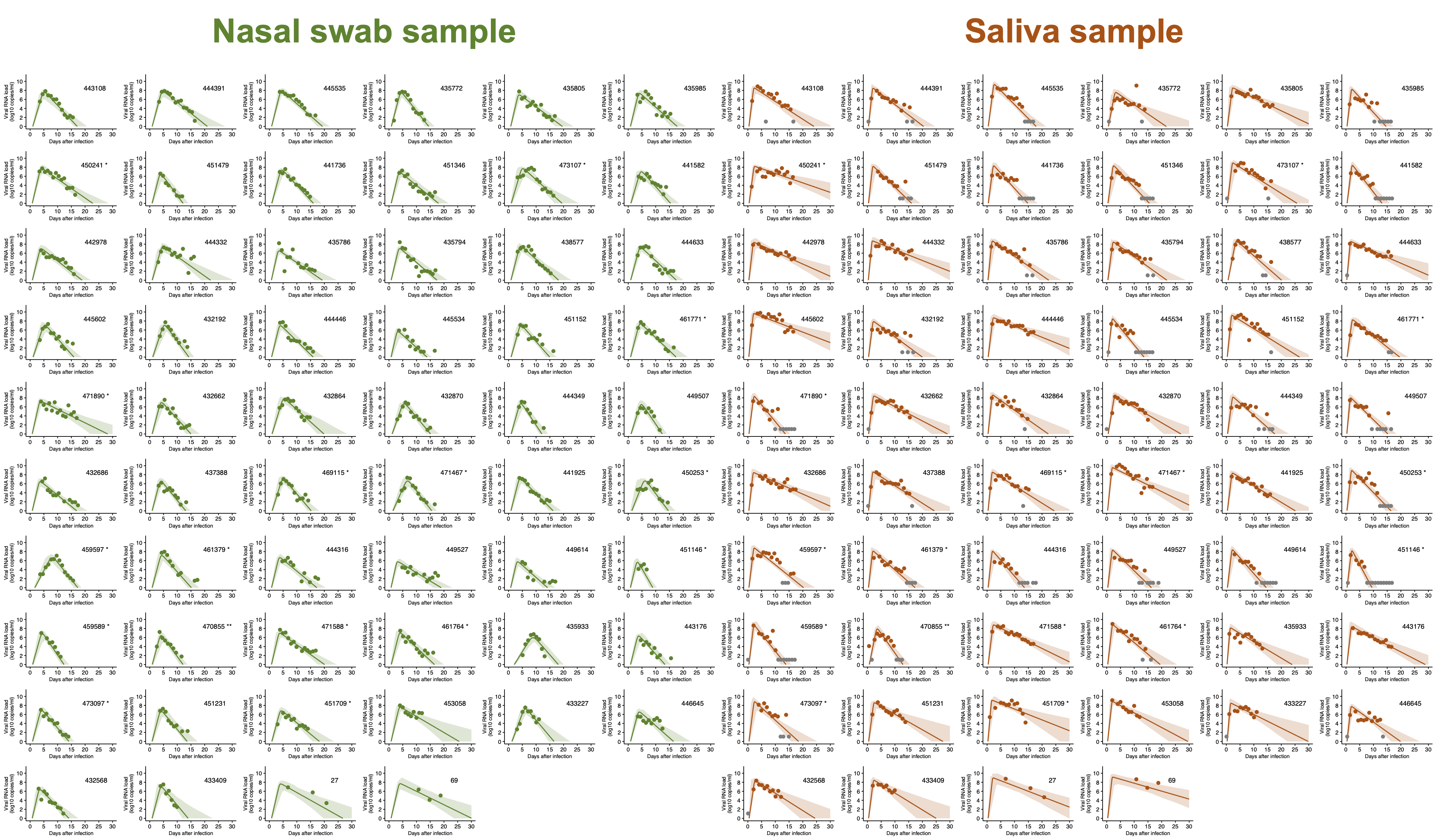


S3 Fig. | Estimated individual viral load trajectory for each COVID-19 case with paired nasal swab and saliva samples: The solid lines are estimated SARS-CoV-2 viral load trajectories under the best-fitting individual parameters. The shaded regions indicate 95% confidence intervals. The green and red dots represent measured viral load observations. The grey dots correspond to observations where the measured viral load was below the limit of detection.
