# Supplementary material for: Identifying the optimal rapid antigen test for screening and determining the end of isolation: A modeling study
Source: PLoS Comput Biol. 2026 Apr 2;22(4):e1013102. doi: 10.1371/journal.pcbi.1013102 (PMC13082731; doi:10.1371/journal.pcbi.1013102)
Supplement: S4 Fig — (A) Risk of transmission during the pre-symptomatic phase, defined as the probability that an infected individual generates at least n secondary cases (≥n) over the screening period. (B) Risk of transmission during the post-symptomatic phase, defined analogously over the isolation period. For each panel, the x-axis shows the secondary case threshold (n=1 or 4; for n=4, we use 1−F(3), where F is the cumulative distribution function) and the y-axis shows the risk of transmission (%). Columns correspond to scenarios with no controls (left), nasal RAT (middle), and saliva RAT (right). The violin plots show the kernel probability density. The box-and-whisker plots show the medians (50th percentile; bold lines), interquartile ranges (25th and 75th percentiles; boxes), and 2.5th to 97.5th percentile ranges (whiskers). Colored violins indicate the distribution of risks under a Poisson offspring model, and gray violins indicate a negative binomial offspring model with dispersion parameter k=0.41. All values were calculated under the baseline settings (screening period = 6 days, full isolation period = 5 days, limit of detection = 6.0 log10 copies/ml, and basic reproduction number R0=3). (DOCX) [file pcbi.1013102.s004.docx]

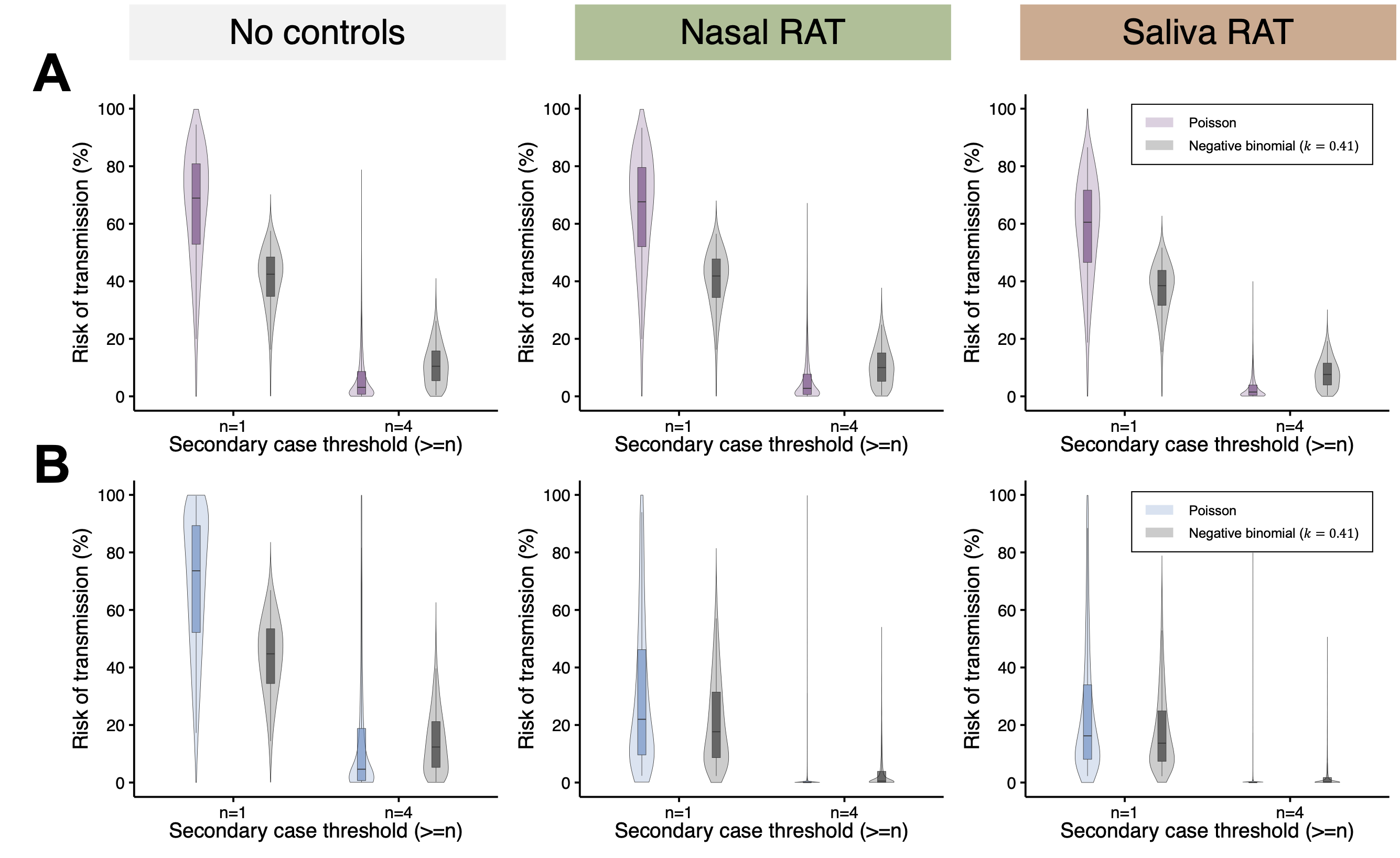


**S4 Fig. | Comparison of risk of transmission under Poisson and negative binomial offspring distributions across control strategies. (A)** Risk of transmission during the pre-symptomatic phase, defined as the probability that an infected individual generates at least $n$ secondary cases ($\geq n$) over the screening period. **(B)** Risk of transmission during the post-symptomatic phase, defined analogously over the isolation period. For each panel, the x-axis shows the secondary case threshold ($n=1$ or $4$; for $n=4$, we use $1-F(3)$, where $F$ is the cumulative distribution function) and the y-axis shows the risk of transmission (%). Columns correspond to scenarios with no controls (left), nasal RAT (middle), and saliva RAT (right). The violin plots show the kernel probability density. The box-and-whisker plots show the medians (50^th^ percentile; bold lines), interquartile ranges (25^th^ and 75^th^ percentiles; boxes), and 2.5^th^ to 97.5^th^ percentile ranges (whiskers). Colored violins indicate the distribution of risks under a Poisson offspring model, and gray violins indicate a negative binomial offspring model with dispersion parameter $k=0.41$ [[2](#_ENREF_2), [3](#_ENREF_3)]. All values were calculated under the baseline settings (screening period $\boldsymbol{= 6}$ days, full isolation period $\boldsymbol{= 5}$ days, limit of detection $\boldsymbol{= 6.0}$ log10 copies/ml, and basic reproduction number $\boldsymbol{R}_{\boldsymbol{0}}\boldsymbol{=3}$).
