# Supplementary material for: Identifying the optimal rapid antigen test for screening and determining the end of isolation: A modeling study
Source: PLoS Comput Biol. 2026 Apr 2;22(4):e1013102. doi: 10.1371/journal.pcbi.1013102 (PMC13082731; doi:10.1371/journal.pcbi.1013102)
Supplement: S5 Fig — Left and right panels show the pre-symptomatic and post-symptomatic phases, respectively. The vertical dashed lines indicate the baseline limit of detection (6.0 log10 copies/ml). The shaded regions correspond to 95% confidence intervals computed using a bootstrap approach. (DOCX) [file pcbi.1013102.s005.docx]

**
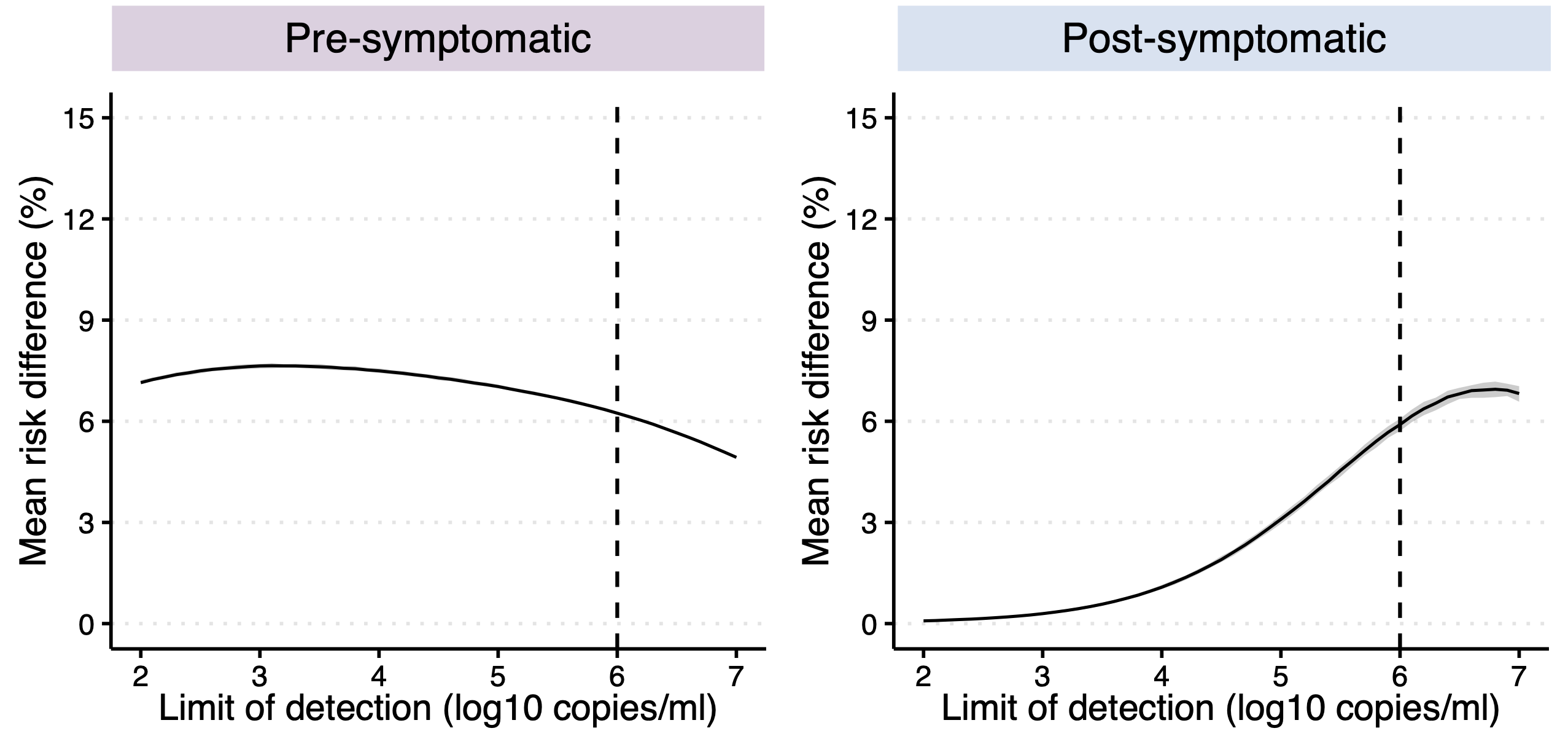
**

S5 Fig. | Sensitivity of the difference in mean risk of transmission between saliva and nasal rapid antigen tests to the limit of detection. Left and right panels show the pre-symptomatic and post-symptomatic phases, respectively. The vertical dashed lines indicate the baseline limit of detection (6.0 log10 copies/ml). The shaded regions correspond to 95% confidence intervals computed using a bootstrap approach.
