# Supplementary material for: Identifying the optimal rapid antigen test for screening and determining the end of isolation: A modeling study
Source: PLoS Comput Biol. 2026 Apr 2;22(4):e1013102. doi: 10.1371/journal.pcbi.1013102 (PMC13082731; doi:10.1371/journal.pcbi.1013102)
Supplement: S6 Fig — (A) Mean risk of transmission after screening with one RAT (i.e., r―pre) in the pre-symptomatic phase. The x-axis and y-axis represent the limit of detection and the screening period, respectively. (B) Mean risk of transmission after ending isolation with RATs (i.e., r―post) in the post-symptomatic phase. The x-axis and y-axis represent the limit of detection and the full isolation period, respectively. All values were calculated under the baseline value of the basic reproduction number (R0=3). The white color regions indicate a risk of 50%. (DOCX) [file pcbi.1013102.s006.docx]

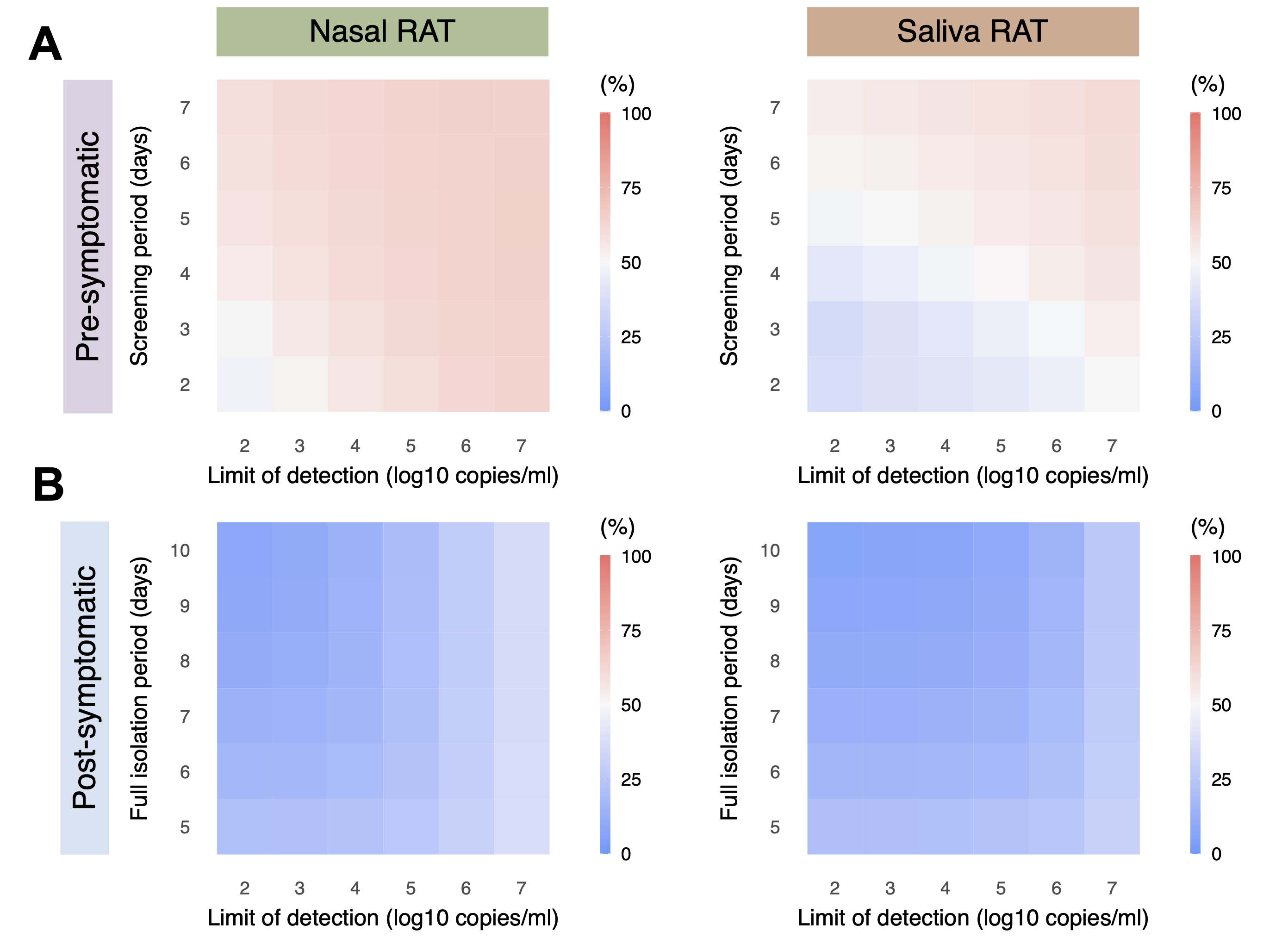


S6 Fig. | Comparison of mean risk of transmission between nasal and saliva rapid antigen tests under different scenarios: (A) Mean risk of transmission after screening with one RAT (i.e., ${\bar{\boldsymbol{r}}}^{\boldsymbol{pre}}$) in the pre-symptomatic phase. The x-axis and y-axis represent the limit of detection and the screening period, respectively. (B) Mean risk of transmission after ending isolation with RATs (i.e., ${\bar{\boldsymbol{r}}}^{\boldsymbol{post}}$) in the post-symptomatic phase. The x-axis and y-axis represent the limit of detection and the full isolation period, respectively. All values were calculated under the baseline value of the basic reproduction number ($\boldsymbol{R}_{\boldsymbol{0}}\boldsymbol{=3}$). The white color regions indicate a risk of $\boldsymbol{50\%}$.
