# Supplementary material for: Identifying the optimal rapid antigen test for screening and determining the end of isolation: A modeling study
Source: PLoS Comput Biol. 2026 Apr 2;22(4):e1013102. doi: 10.1371/journal.pcbi.1013102 (PMC13082731; doi:10.1371/journal.pcbi.1013102)
Supplement: S7 Fig — The x-axis and y-axis represent days after symptom onset and the value of the basic reproduction number (i.e., R0), respectively. (B) Mean risk of transmission after screening with one RAT (i.e., r―pre) in the pre-symptomatic phase. All values were calculated under the baseline settings (screening period = 6 days and limit of detection = 6.0 log10 copies/ml). (C) Mean risk of transmission after ending isolation with RATs (i.e., r―post) in the post-symptomatic phase. All values were calculated under the baseline settings (full isolation period = 5 days and limit of detection = 6.0 log10 copies/ml). The shaded regions correspond to 95% confidence intervals computed using a bootstrap approach. The black vertical dashed lines indicate the baseline value of the basic reproduction number (R0=3). (DOCX) [file pcbi.1013102.s007.docx]

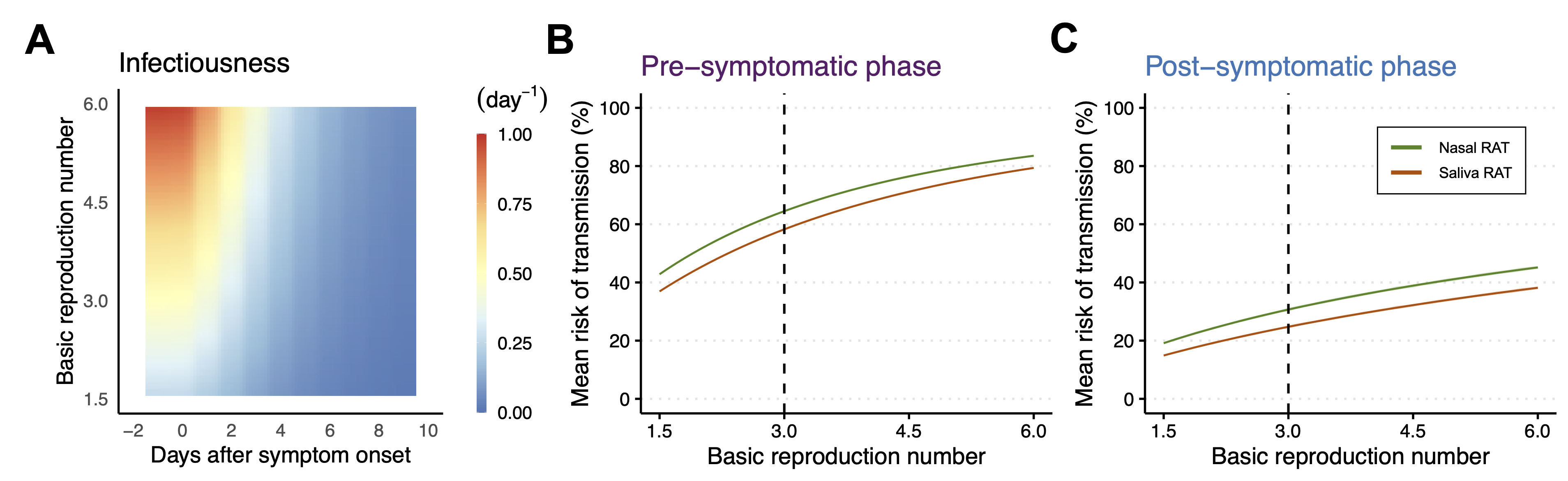


S7 Fig. | Comparison of risk of transmission between using nasal and saliva rapid antigen tests under different values of basic reproduction number: (A) Estimated mean infectiousness in the absence of controls. The x-axis and y-axis represent days after symptom onset and the value of the basic reproduction number (i.e., $\boldsymbol{R}_{\boldsymbol{0}}$), respectively. (B) Mean risk of transmission after screening with one RAT (i.e., ${\bar{\boldsymbol{r}}}^{\boldsymbol{pre}}$ ) in the pre-symptomatic phase. All values were calculated under the baseline settings (screening period $\boldsymbol{= 6}$ days and limit of detection $\boldsymbol{= 6.0}$ log10 copies/ml). (C) Mean risk of transmission after ending isolation with RATs (i.e., ${\bar{\boldsymbol{r}}}^{\boldsymbol{post}}$ ) in the post-symptomatic phase. All values were calculated under the baseline settings (full isolation period $\boldsymbol{= 5}$ days and limit of detection $\boldsymbol{= 6.0}$ log10 copies/ml). The shaded regions correspond to 95% confidence intervals computed using a bootstrap approach. The black vertical dashed lines indicate the baseline value of the basic reproduction number ($\boldsymbol{R}_{\boldsymbol{0}}\boldsymbol{=3}$).
