# Supplementary material for: Identifying the optimal rapid antigen test for screening and determining the end of isolation: A modeling study
Source: PLoS Comput Biol. 2026 Apr 2;22(4):e1013102. doi: 10.1371/journal.pcbi.1013102 (PMC13082731; doi:10.1371/journal.pcbi.1013102)
Supplement: S8 Fig — Left and right panels show the pre-symptomatic and post-symptomatic phases, respectively. The vertical dashed lines indicate the baseline value of the basic reproduction number (R0=3). The shaded regions correspond to 95% confidence intervals computed using a bootstrap approach. (DOCX) [file pcbi.1013102.s008.docx]

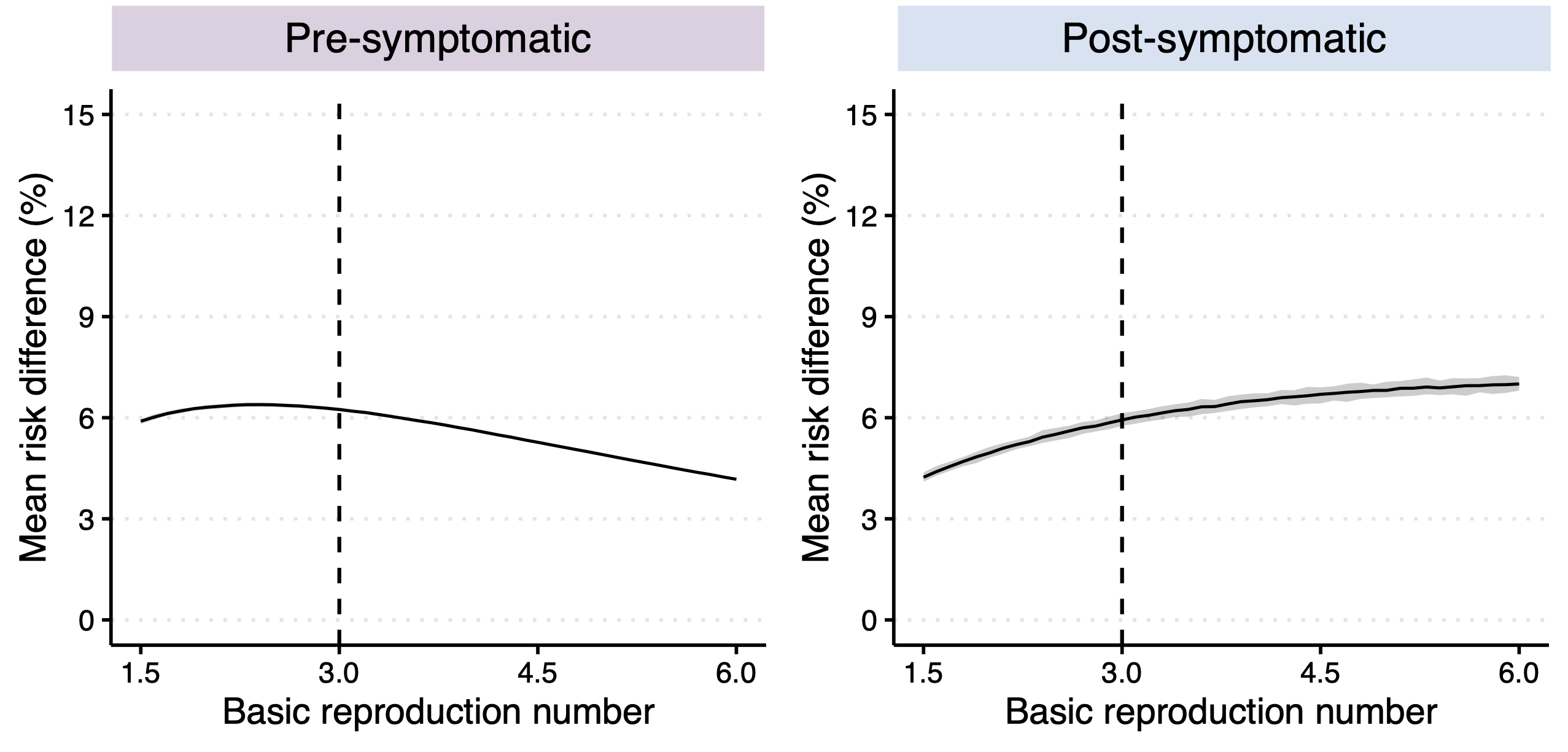


S8 Fig. | Sensitivity of the difference in mean risk of transmission between saliva and nasal rapid antigen tests to the basic reproduction number. Left and right panels show the pre-symptomatic and post-symptomatic phases, respectively. The vertical dashed lines indicate the baseline value of the basic reproduction number ($\boldsymbol{R}_{\boldsymbol{0}}\boldsymbol{=3}$). The shaded regions correspond to 95% confidence intervals computed using a bootstrap approach.
