# Supplementary material for: Identifying the optimal rapid antigen test for screening and determining the end of isolation: A modeling study
Source: PLoS Comput Biol. 2026 Apr 2;22(4):e1013102. doi: 10.1371/journal.pcbi.1013102 (PMC13082731; doi:10.1371/journal.pcbi.1013102)
Supplement: S9 Fig — (A) Mean risk of transmission after screening with one RAT (i.e., r―pre) in the pre-symptomatic phase. The values were calculated under the baseline settings (screening period = 6 days and limit of detection = 6.0 log10 copies/ml). (B) Mean risk of transmission after ending isolation with RATs (i.e., r―post) in the post-symptomatic phase. The values were calculated under the baseline settings (full isolation period = 5 days and limit of detection = 6.0 log10 copies/ml). All values were calculated under the baseline value of the basic reproduction number (R0=3). (DOCX) [file pcbi.1013102.s009.docx]

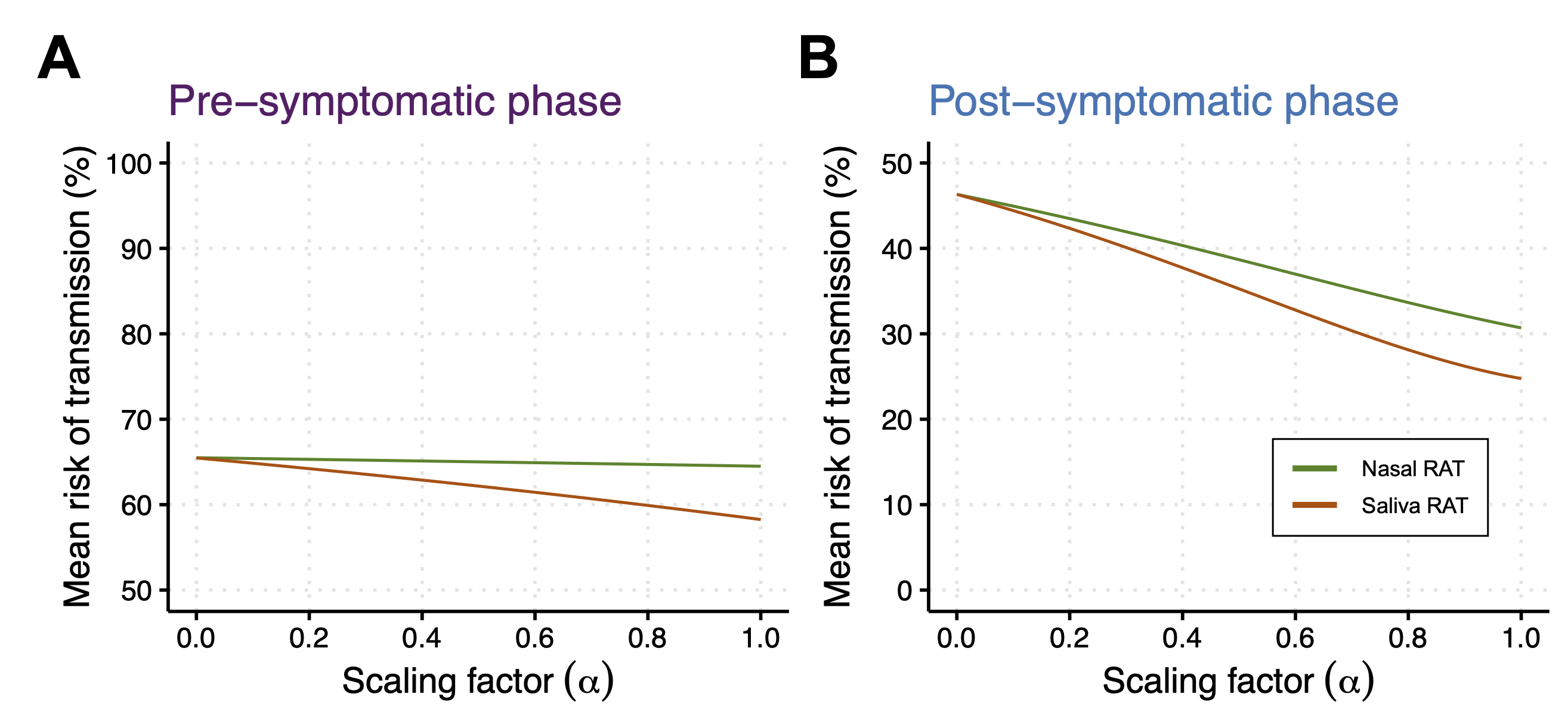


**S9 Fig. | Comparison of mean risk of transmission between nasal and saliva rapid antigen tests under different values of scaling factor:** **(A)** Mean risk of transmission after screening with one RAT (i.e., ${\bar{\boldsymbol{r}}}^{\boldsymbol{pre}}$ ) in the pre-symptomatic phase. The values were calculated under the baseline settings (screening period $\boldsymbol{= 6}$ days and limit of detection $\boldsymbol{= 6.0}$ log10 copies/ml). **(B)** Mean risk of transmission after ending isolation with RATs (i.e., ${\bar{\boldsymbol{r}}}^{\boldsymbol{post}}$ ) in the post-symptomatic phase. The values were calculated under the baseline settings (full isolation period $\boldsymbol{= 5}$ days and limit of detection $\boldsymbol{= 6.0}$ log10 copies/ml). All values were calculated under the baseline value of the basic reproduction number ($\boldsymbol{R}_{\boldsymbol{0}}\boldsymbol{=3}$).
