# Supplementary material for: Identifying the optimal rapid antigen test for screening and determining the end of isolation: A modeling study
Source: PLoS Comput Biol. 2026 Apr 2;22(4):e1013102. doi: 10.1371/journal.pcbi.1013102 (PMC13082731; doi:10.1371/journal.pcbi.1013102)
Supplement: S10 Fig — The expected isolation period under saliva RATs (right panel). The x-axis and y-axis represent the limit of detection and the full isolation period, respectively. All values were calculated under the baseline value of the basic reproduction number (R0=3). (DOCX) [file pcbi.1013102.s010.docx]

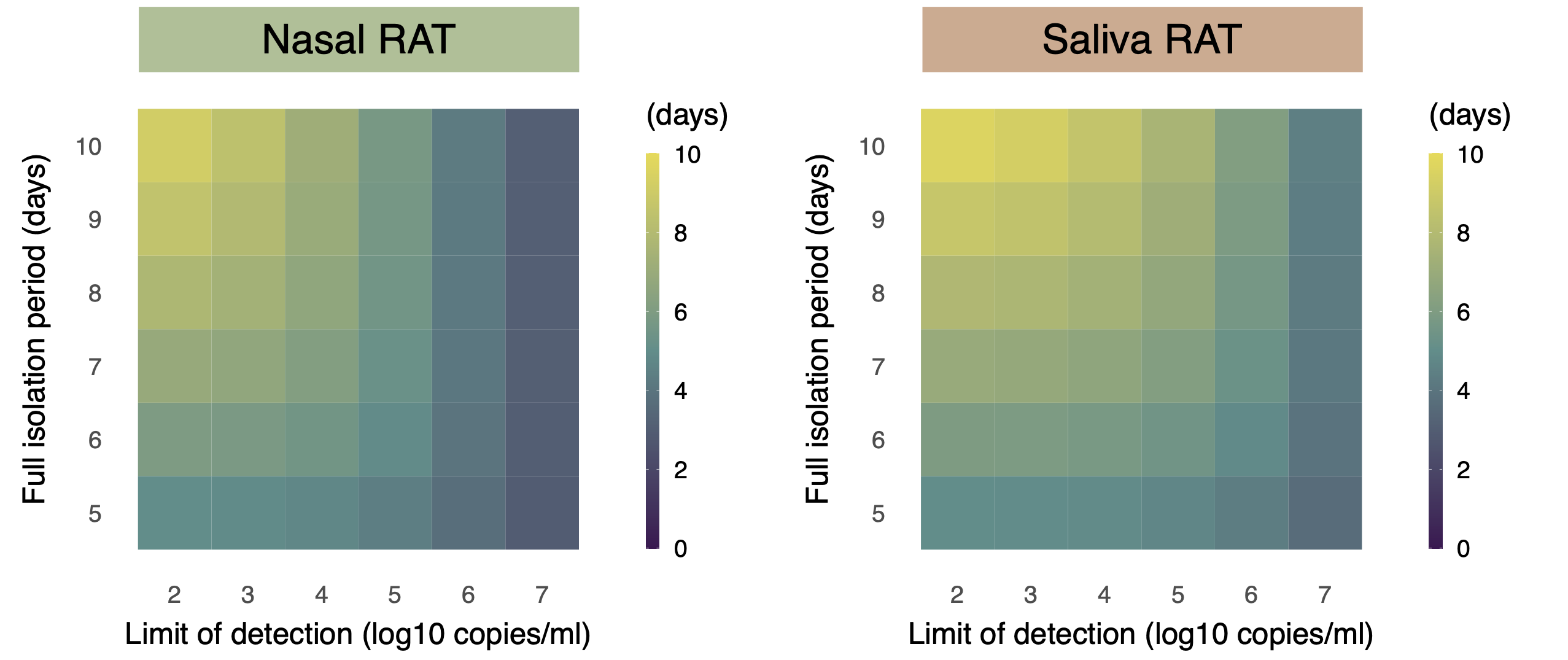


S10 Fig. | Evaluation of ending isolation with RATs under different scenarios in the post-symptomatic phase: The expected isolation period under nasal RATs (left panel). The expected isolation period under saliva RATs (right panel). The x-axis and y-axis represent the limit of detection and the full isolation period, respectively. All values were calculated under the baseline value of the basic reproduction number ($\boldsymbol{R}_{\boldsymbol{0}}\boldsymbol{=3}$).
