# Supplementary material for: Identifying the optimal rapid antigen test for screening and determining the end of isolation: A modeling study
Source: PLoS Comput Biol. 2026 Apr 2;22(4):e1013102. doi: 10.1371/journal.pcbi.1013102 (PMC13082731; doi:10.1371/journal.pcbi.1013102)
Supplement: S1 Table — (DOCX) [file pcbi.1013102.s011.docx]

S1 Table. | Summary of SARS-CoV-2 viral load data with paired nasal swab and saliva samples

| **Country** | **Number of individuals** | **Reporting unit** | **Source** |
| --- | --- | --- | --- |
| USA | $56$ | cycle threshold^#^ | [[4](#_ENREF_4)] |
| USA | $2$ | viral load (copies/ml) | [[5](#_ENREF_5)] |

^#^ Viral loads in nasal and saliva swab samples were calculated using different conversion formulas, respectively: $\log_{10} \left( \text{Viral RNA load [copies/ml]} \right)=11.35-0.25\times\text{Ct values [cycles]}$ and $\log_{10} \left( \text{Viral RNA load [copies/ml]} \right)=14.24-0.28\times\text{Ct values [cycles]}$ for nasal swab samples and saliva samples, respectively [[4](#_ENREF_4)].
