# Supplementary material for: Identifying the optimal rapid antigen test for screening and determining the end of isolation: A modeling study
Source: PLoS Comput Biol. 2026 Apr 2;22(4):e1013102. doi: 10.1371/journal.pcbi.1013102 (PMC13082731; doi:10.1371/journal.pcbi.1013102)
Supplement: S2 Table — (DOCX) [file pcbi.1013102.s012.docx]

S2 Table. | Estimated fixed effect parameters, standard deviation of random effects, and standard deviation of error in SARS-CoV-2 log viral loads from nasal swab and saliva samples

| **Parameters** | **Symbol** | **Unit** | **Nasal swab sample** | | **Saliva sample** | |
| --- | --- | --- | --- | --- | --- | --- |
|  |  |  | Fixed effect | SD of random effect | Fixed effect | SD of random effect |
| Maximum rate constant for viral replication | $\gamma$ | day^-1^ | $6.90$ | $0.13$ | $13.29$ | $0.28$ |
| Rate constant for virus infection | $b$ | (copies/ml)^-1^day^-1^ | ${3.52\times10}^{-7}$ | $1.59$ | ${3.18\times10}^{-8}$ | $1.37$ |
| Death rate of infected cells | $\delta$ | day^-1^ | $1.49$ | $0.43$ | $0.95$ | $0.47$ |
| Incubation period | $\tau$ | day | $4.07$ | $0.18$ | $4.07$ | $0.18$ |
| Standard deviation of error | $\sigma$ | log10 copies/ml | $1.31$ | | $1.24$ | |
