# Supplementary material for: Identifying the optimal rapid antigen test for screening and determining the end of isolation: A modeling study
Source: PLoS Comput Biol. 2026 Apr 2;22(4):e1013102. doi: 10.1371/journal.pcbi.1013102 (PMC13082731; doi:10.1371/journal.pcbi.1013102)
Supplement: S3 Table — (DOCX) [file pcbi.1013102.s013.docx]

S3 Table. | Parameter values for modeling transmissibility of SARS-CoV-2

| **Parameters** | **Symbol** | **Value** | **Source** |
| --- | --- | --- | --- |
| Half-saturation constant | $K_{m}$ | $2.72\times{10}^{8}$ | Fitted |
| Hill coefficient | $h$ | $0.5471$ | Fitted |
| Basic reproduction number | $R_{0}$ | $3.0 (1.5-6.0)$ | [[6-10](#_ENREF_6)] |
